# Training of ultra-fast speech comprehension induces functional reorganization of the central-visual system in late-blind humans

**DOI:** 10.3389/fnhum.2013.00701

**Published:** 2013-10-23

**Authors:** Susanne Dietrich, Ingo Hertrich, Hermann Ackermann

**Affiliations:** Department of General Neurology, Center for Neurology, Hertie Institute for Clinical Brain Research, University of TübingenTübingen, Germany

**Keywords:** cross-modal plasticity, speech perception, residual vision, strategies, blindness

## Abstract

Individuals suffering from vision loss of a peripheral origin may learn to understand spoken language at a rate of up to about 22 syllables (syl) per seconds (s)—exceeding by far the maximum performance level of untrained listeners (ca. 8 syl/s). Previous findings indicate the central-visual system to contribute to the processing of accelerated speech in blind subjects. As an extension, the present training study addresses the issue whether acquisition of ultra-fast (18 syl/s) speech perception skills induces de novo central-visual hemodynamic activation in late-blind participants. Furthermore, we asked to what extent subjects with normal or residual vision can improve understanding of accelerated verbal utterances by means of specific training measures. To these ends, functional magnetic resonance imaging (fMRI) was performed while subjects were listening to forward and reversed sentence utterances of moderately fast and ultra-fast syllable rates (8 or 18 syl/s) prior to and after a training period of ca. 6 months. Four of six participants showed—independently from residual visual functions—considerable enhancement of ultra-fast speech perception (about 70% points correctly repeated words) whereas behavioral performance did not change in the two remaining participants. Only subjects with very low visual acuity displayed training-induced hemodynamic activation of the central-visual system. By contrast, participants with moderately impaired or even normal visual acuity showed, instead, increased right-hemispheric frontal or bilateral anterior temporal lobe responses after training. All subjects with significant training effects displayed a concomitant increase of hemodynamic activation of left-hemispheric SMA. In spite of similar behavioral performance, trained “experts” appear to use distinct strategies of ultra-fast speech processing depending on whether the occipital cortex is still deployed for visual processing.

## Introduction

The acquisition of sensorimotor or perceptual skills is associated with functional brain reorganization, and these processes may emerge within a single or extend across several modality-specific cortical regions. For example, training-induced neuroplasticity of auditory association areas has been observed in normal-sighted subjects who learned to understand time-compressed speech (Adank and Devlin, 2010). By contrast, cross-modal mechanisms appear to contribute to the processing of non-visual (tactile or auditory) stimuli or language-related tasks in blind individuals. Striate cortex, e.g., has been found to show significant hemodynamic activation during Braille reading (e.g., Büchel et al., 1998; Sadato et al., 1998; Gizewski et al., 2003; Sadato, 2005; Burton et al., 2006), auditory motion detection (Poirier et al., 2006), syntactic and semantic speech processing (Röder et al., 2002), verb generation, production of mental images based upon animal names, and verbal episodic memory retrieval (Amedi et al., 2003; Lambert et al., 2004; Raz et al., 2005). As a further example, vision-impaired individuals may learn to understand accelerated spoken language at a rate of up to about 22 syllables (syl) per second (s)—exceeding by far the capacities of normal sighted listeners whose upper limit is at ca. 8 syl/s (Moos and Trouvain, 2007). This exceptional skill allows for the processing of large amounts of written materials using screen-reading text-to-speech devices and may help, e.g., to better cope with the demands of college or university education. As a first approach to the elucidation of the cerebral mechanisms underlying these intriguing perceptual/cognitive capacities, a previous fMRI study of our group (Dietrich et al., 2013) delineated the hemodynamic activation pattern of late-blind and sighted individuals while listening to sentence utterances of a moderately fast (8 syl/s) or ultra-fast (16 syl/s) syllable rate. The proficiency of the blind subjects extended from low to high comprehension capabilities (up to >90%—in terms of the percentage of syllables in correctly reproduced words in a sentence repetition task) at 16 syl/s, whereas the performance level of sighted subjects fell consistently below 20%. Besides the classical perisylvian “language zones” of the left hemisphere [inferior frontal gyrus (IFG)/superior temporal cortex] and the supplementary motor area (SMA), blind people highly skilled in ultra-fast speech perception showed significant hemodynamic activation of right-hemispheric primary visual cortex (V1), contralateral fusiform gyrus (FG), and bilateral pulvinar (Pv) (Dietrich et al., 2013). In a recent Hypothesis and Theory paper (Hertrich et al., 2013b), an expanded model of speech perception was introduced to describe how blind subjects might use their visual system for ultra-fast speech perception. Thereby, right visual cortex enhances time-critical speech processing due to its cross-links to (i) the afferent auditory pathway (e.g., via Pv) and (ii) to frontal action-related representations (e.g., SMA, IFG). Experimental data have shown that the visual system can impact auditory perception at basic computational stages such as temporal signal resolution. For instance, magnetoencephalographic measurements revealed an early field component in right occipital cortex phase-locked to the syllable onsets of accelerated speech (Hertrich et al., 2013a). In normal sighted people, the “bottleneck” for understanding time-compressed speech seems related to higher demands on the buffering of phonological materials and is presumably linked to frontal brain structures. Thus, occipito-frontal interaction via SMA might be an important factor for overcoming this bottleneck.

Moos and Trouvain (2007) as well as our previous group study (Dietrich et al., 2013) used as participants a group of blind performers and normal sighted non-performers and, thus, the factors *blindness* and *performance* were confounded. Thus, the question whether vision might be a limitation for ultra-fast speech comprehension is not yet answered. Training studies can be expected to further elucidate the relationship between neuroimaging data and behavioral performance during ultra-fast speech perception. More specifically, we hypothesized—based upon our preceding work—that, first, the acquisition of ultra-fast speech comprehension skills translates into hemodynamic activation of the visual system, indicating that enhanced spoken language processing might be associated with the recruitment of occipital cortex. Second, the strength of training-induced responses of the visual system was expected to parallel the extent of vision impairment. Third, activation of left IFG and SMA—as components of inner speech representations—might reflect speech understanding. In particular, left SMA is hypothesized to play a role in coordinating prosodic features such as the timing of syllable onsets with phonetic representations in IFG which is also assumed to strongly interact with the mental lexicon. The data, furthermore, will be interpreted in terms of different neuroplasticity patterns as suggested by Kelly and Garavan (2005), i.e., the distinction between reorganization and redistribution during the acquisition of a new skill. Thereby, functional reorganization—in terms of recruiting an additional area with training associated with the shift in the cognitive processes underlying performance (see also Guida et al., 2012)—is expected in blind subjects with strongly reduced vision. By contrast, subjects with residual vision after training may just increase activation within the already existing classical language network.

As a first and still preliminary test of these suggestions, five late-blind subjects varying in residual vision capacities and one normal sighted individual—all of them never exposed to accelerated spoken language before—were instructed to train ultra-fast speech comprehension capacities over a period of ca. 6 months. The participants underwent behavioral performance tests as well as fMRI measurements prior to and after the training sessions while listening to sentence utterances of a moderately fast (8 syl/s) and ultra-fast (18 syl/s) speaking rate. As a control condition, the same test materials were applied as time-reversed events to the participants, representing unintelligible signals of a matched distribution of spectral energy. Given the relatively small sample size of the training study, each of the six individuals (differing in their residual vision) was evaluated based on whole-brain analyses as a single case. Furthermore, the “activation spots” of our previous group study (Dietrich et al., 2013) which displayed a significant covariance between BOLD responses and ultra-fast speech perception capabilities (right V1, ipsilateral Pv, left SMA, and left IFG) were considered regions-of-interest (ROI) and analyzed in more detail at the group level.

## Materials and methods

### Participants

Five blind subjects and a single normal-sighted individual (3 males; mean age = 34.3 years, *SD* = 11.99) participated in this functional imaging experiment (Table 1). All of them were right-handed (Edinburgh handedness inventory) native German speakers without a history of neurological problems or hearing deficits as determined by means of an audiogram. The study design had been approved by the ethics committee of the University of Tübingen. All blind participants received a set of written information (MRI guidelines, data protection, and consent form) as pdf-files by email. Prior to fMRI measurements, the experimenter read, in addition, the materials aloud to each blind individual, and the consent form was signed in the presence of a sighted witness. Since the blind participants were recruited from community organizations, a detailed clinical data bank was not available to the authors and, thus, information on etiology and follow-up of the ophthalmological disorders had to be drawn from personal interviews and previous medical records. In all instances, a peripheral origin of blindness could be established, but the participants represented a rather heterogeneous group with respect to residual vision capabilities, i.e., peripheral visual field and visual acuity (see Table 1). The evaluation of the hemodynamic response patterns of each participant, therefore, had to take into account his/her individual profile of visual functions. Three of the six participants (see Table 1) showed no or low visual capabilities (nos. 147, 151, 150), residual or normal functions (nos. 144, 146, 142).

**Table 1:** **Clinical and behavioral data of the vision-impaired and healthy subjects**.

**Subject**	**Behavioral performance (%)**						**Etiology**			
	**Pre-training**			**Post-training**			**Visual acuity (%)**		**Peripheral visual field**	**Cause of blindness**
	**8 syl/s**	**13 syl/s**	**18 syl/s**	**8 syl/s**	**13 syl/s**	**18 syl/s**	**Left**	**Right**		
147	100	89	0	83	97	68	<2	<2	None	Retinitis pigmentosa since age of 7, severely deteriorated after the age of 36 years
151	100	56	0	100	82	36	<2	<2	Intact	Macular degeneration (dystrophy of rods and cones) since age of 29
150	100	62	0	100	97	70	2	10	Reduced	Myopia (short-sightedness) severely deteriorated after the age of 45 years
144	100	69	8	100	93	79	20	5	Reduced	Retinitis pigmentosa since age of 13
146	100	80	14	100	92	14	50	50	Reduced	Retinitis pigmentosa since age of 3
142	100	56	0	100	96	67	Normal-sighted			

### Training procedure

After a baseline session (behavioral testing and fMRI measurements), the participants were instructed to use the screen-reader JAWS (male voice, synthesizer “Eloquence,” http://www.freedomsci.de) for at least 1 h per day. They received digital newspapers on a regular basis, but might also “read” other texts, e.g., e-books. Furthermore, the subjects were encouraged to speed up more and more the syllable rate according to their actual training level. When they themselves had the impression to understand more than 80% of the texts at a level of 13 syl/s, they were invited to a second series of fMRI measurements (i.e., first training target). A third functional imaging session was conducted as soon as a syllable rate of 18 syl/s (final training target) could be mastered. The time span between the recording sessions amounted to ca. 3 months, but could ultimately be determined by the subjects themselves. The present study primarily compares the pre- (baseline) and post-training (trained at 18 syl/s) measurements, whereas the results of the intermediate session (trained at 13 syl/s), including complex de-/activation patterns, are not in the focus of the discussion.

### Stimuli of the fMRI experiment

Three sets (to be used at different sessions) of 90 different text passages comprising one or two sentences each were collected from newspapers. All these test items had a duration of ca. 4 s after text-to-speech conversion (formant synthesizer “eloquence” implemented in the synthesizer JAWS), at the three speaking rates considered (30 stimuli each at 8, 13, or 18 syl/s; see Supplementary files 1, 2 for examples). Thus, the faster test sentences encompassed more text than the slower ones. In a calibration experiment prior to the training study, the relationship between the internal speed parameters of the JAWS system and the respective mean syllable rate was determined. In addition, all stimuli were converted into time-reversed speech signals, serving as spectrally matched, but unintelligible control items (Supplementary files 3, 4). Altogether, thus, each stimulus set contained 30 (items) × 3 (rates) × 2 (forward/backward) = 180 stimuli that were presented in pseudo-randomized order within one session.

### Behavioral data acquisition and analysis

To obtain a quantitative behavioral measure of an individual's capability to understand time-compressed speech, each subject performed—outside the scanner and prior to the fMRI measurements—a sentence repetition task, encompassing a set of 33 sentences of a length of 18 syllables each. These verbal utterances were played to the participants at different speaking rates amounting from 6 syl/s up to 22 syl/s, using a manual staircase procedure in order to determine the actual syllable rate at which repetition performance amounted to about 80% (total percentage of syllables across all correctly repeated words). The stimulus materials were presented to the participants via a loudspeaker (Fostex, 6301B) within a sound-attenuated room, subjects being asked to repeat them “as good as possible,” even when they failed to “grasp” all words (see Supplementary file 5 for an example). The subjects' repetitions were digitally recorded (M-audio Microtrack 2496) and underwent subsequent quantitative evaluation of speech comprehension (percentage of correctly reproduced syllables (see above), ignoring minor errors such as plural endings). Admittedly, this repetition task has a memory component that might interfere with spoken language intelligibility. However, in a previous study (Dietrich et al., 2013) we observed a strong ceiling effect for moderately fast speech, indicating that memory is not the limiting factor in the subjects' performance.

### fMRI—data acquisition

Based upon an event-related design, the set of 180 stimuli of each functional imaging session (30 items at three speaking rates plus the respective backward signals) as well as 40 silent baseline events (scanner noise only) were applied in a randomized order, subdivided into five runs of 44 stimuli each as binaural-symmetric signals to the participants via headphones (Sennheiser HD 570; modified by removal of the permanent magnet, see Baumgart et al., 1998). Since these headphones show sufficient dampening of environmental noise, it was not necessary to provide additional earplugs during the experiments. The inter-stimulus interval amounted to 9.6 s (jitter ± 1.4 s, steps of 0.2 s). Since the design did not allow for an explicit control of speech comprehension during the fMRI experiment, behavioral performance was evaluated offline, and during scanning subjects were just instructed to listen carefully to the applied auditory stimuli and to try to understand them. Thus, the design did not allow for an explicit control of speech comprehension during the fMRI experiment. However, the brain structures sensitive to speech intelligibility have been found to “light up” even under listening-only conditions (Scott et al., 2000). And activation of language processing areas such as IFG has been considered an indicator of actual speech comprehension (Poldrack et al., 2001). Subjects were asked to close their eyes during scanning and to report to the experimenters whether they could adequately hear the test materials in the presence of scanner noise, otherwise the sound amplitude of the stimuli was further adjusted.

The experiment was run on a 3 Tesla MRI system (Magnetom TRIO; Siemens, Erlangen, Germany), using an echo-planar imaging sequence (echo-time = 30 ms, 64 × 64 matrix with a resolution of 3 × 3 mm^2^, 27 axial slices across the whole brain volume, TR = 1.6 s, slice thickness = 4 mm, flip angle = 90°, 270 scans per run). The scanner generated a constant background noise throughout fMRI measurements, serving as the baseline condition of the experimental design during the null events. Anatomical images required for the localization of the hemodynamic responses were obtained by means of a MDEFT sequence (T1-weighted images, TR = 2.3 s, TE = 2.92 ms, flip angle = 8°, slice thickness = 1 mm, resolution = 1 × 1 mm^2^) of a bi-commissural (AC-PC) orientation.

### fMRI—data analyses

Preprocessing of the data encompassed slice time and motion correction, normalization to the Montreal Neurological Institute (MNI) template space, and smoothing by means of an 8 mm full-width half-maximum Gaussian kernel (SPM5 software package; http://www.fil.ion.ucl.ac.uk/spm). For the sake of statistical analysis, the blood oxygen level-dependent (BOLD) responses were modeled by means of a prototypical hemodynamic function within the context of a general linear model, event durations being specified as 4 s epochs. Any low-frequency temporal drifts were removed using a 128 s high-pass filter.

The evaluation of the functional imaging data encompassed the following steps of signal analysis:

1. In order to delineate the brain regions engaged in the processing of moderately fast (=8 syl/s) and ultra-fast (=18 syl/s) verbal utterances before and after the learning interval, pre- and post-training runs were analyzed in a concatenated sequence (1st level SPM analysis). First, the SPM *T*-contrast of hemodynamic activation vs. baseline (and vice versa) was computed separately for each subject (the SPM coordinates resulting from the contrasts are listed in Supplementary files 6, 7). Second, direct comparisons between post- and pre-training fMRI data during the ultra-fast speech condition (not vs. baseline) as well as during the baseline (=null-event) were additionally analyzed separately for each subject (the SPM coordinates resulting from the contrasts are listed in Supplementary files 8, 9). Whole-brain SPM *T*-contrasts were threshold at *p* < 0.005 uncorrected at voxel level with an extent threshold *k* = 10 voxels.
2. For the evaluation of the interaction of the experimental conditions considered (vs. baseline), a whole-brain ANOVA including the factors *Mode* (forward/backward speech), *Speech rate* (8/18 syl/s), and *Training* (pre-/post-training) was conducted within two single subjects—no. 147 with no residual vision and no. 142 normal sighted both with significant training effects (whole-brain full factorial ANOVA, threshold *p* < 0.005 uncorrected at voxel level, extent threshold *k* = 10 voxels; the SPM coordinates resulting from the three-way interaction *Mode* × *Speech rate* × *Training* are listed in Supplementary file 10).
3. Additionally, it was hypothesized that primary visual cortex activation depends on low residual vision. Thus, a whole-brain covariance analysis was performed with residual vision as a rank covariate (SPM *T*-contrast “post- minus pre-training” regarding the forward ultra-fast speech condition, calculated across the six subjects; whole-brain one-sample *T*-test, threshold *p* < 0.005 uncorrected at voxel level, extent threshold *k* = 10 voxels).
4. In order to obtain quantitative data for a comparison of the six participants with each other, four activation clusters (see Figure 4) hypothesized to be strongly related to ultra-fast speech comprehension capabilities were selected from our previous group study of late-blind subjects (Dietrich et al., 2013): Right V1 activation was considered an index of visual system recruitment (peak coordinates = 15, −102, 6; cluster size = 368 voxels), ipsilateral Pv (18, −30, 6; 23 voxels) had emerged as an input region projecting toward right V1 via the secondary visual pathway, left-hemispheric SMA (−6, 9, 60; 112 voxels) may serve as a potential interface between syllabic event timing and inner speech representations, and left-hemispheric IFG (−48, 18, 18; 162 voxels) seems to be involved in the phonological encoding of inner speech representations. Against the background of our model of ultra-fast speech understanding derived from previous work (Hertrich et al., 2013b), only blind, but not sighted subjects were expected to recruit the central-visual system whereas, by contrast, participants with residual or normal vision should deactivate the visual-system during listening to speech. As concerns SMA, already identified as a “bottleneck” of time-compressed spoken language perception (Vagharchakian et al., 2012), hemodynamic activation was expected (i) to reflect gains in behavioral performance and (ii) to increase with speech rate in high-performing subjects. As a control, hemodynamic IFG activation during ultra-fast speech perception is also hypothesized to increase with training state, but will not show comparable effects that are directly related to speech rate. Spearman's rank correlation analysis (coefficient ρ) was applied to test for the impact of residual vision upon BOLD responses (rank 1–6, 1 = no residual vision, 6 = normal sighted) as well as Pearson's correlation (coefficient *r*) to evaluate the relation between individual behavioral performance and BOLD responses pre- and post-training. Spearman's rank correlation was chosen because the relationship between residual vision and BOLD responses of the visual system is not expected to be linear (compared to a more linear relationship between behavioral performance and IFG/SMA activation). Furthermore, *T* statistic was used testing individual de- or activation patterns, differences between forward and reversed speech conditions, and differences between the slopes of regression lines corresponding to pre- vs. post-training data. Since our hypotheses make predictions regarding the direction of effects, one-sided statistical tests were applied.

## Results

### Behavioral performance after the training

Prior to the training procedure, none of the subjects was able to understand ultra-fast speech at a level of 18 syl/s. More specifically, the percentage of correctly reproduced syllables fell below 14% in all instances. At (the second) follow-up examination, four subjects (nos. 147, 150, 144, and 142) showed an improved behavioral performance of up to ca. 70% points (mean = 71%, *SE* = 2.74%). This subgroup included participants with low or high residual or even normal vision. A single participant (no. 146) failed to show any learning effects, and another one (no. 151) achieved only modest accomplishments amounting to about 36% points (Table 1). Regarding moderately fast (8 syl/s) and accelerated speech at 13 syl/s, after training all subjects performed with >80% (8 syl/s: mean = 97%, *SE* = 6.94%; 13 syl/s: mean = 93%, *SE* = 5.71%) correctly reproduced syllables (Table 1).

### Whole-brain analyses

#### Ultra-fast speech vs. baseline pre-training

**Figure 1:**
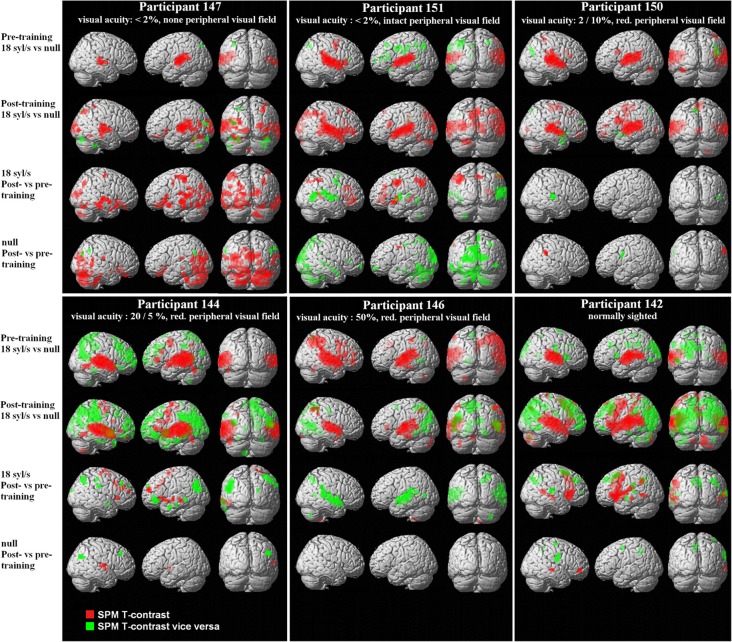
**Whole-brain analyses from six single subjects differing in residual vision, based upon the SPM *T*-contrasts for forward ultra-fast speech vs. baseline pre- and post-training (red = condition vs. baseline, green = baseline vs. condition), the direct contrast post- vs. pre-training with respect to the ultra-fast condition as well as the training-induced baseline shift in the null-event (red = post- vs. pre-training, green = pre- vs. post-training).** Threshold at *p* < 0.005 uncorrected with an extent threshold *k* = 10 voxels (for peak coordinates see Supplementary files 6–9).

Prior to the training period, hemodynamic responses to ultra-fast verbal utterances emerged within primary auditory areas of either hemisphere as well as adjacent structures of the superior/middle temporal cortex in all subjects (SPM *T*-contrast “forward ultra-fast speech vs. baseline,” *p* < 0.005 uncorrected with an extent threshold *k* = 10 voxels; Figure 1; Supplementary file 6). Additionally, some participants showed activation within left- and right-hemispheric frontal, and parietal regions, the left cerebellum, and subcortical supratentorial areas such as the hippocampus, thalamus, or pallidum (Supplementary file 6).

#### Ultra-fast speech vs. baseline post-training

**Figure 2:**
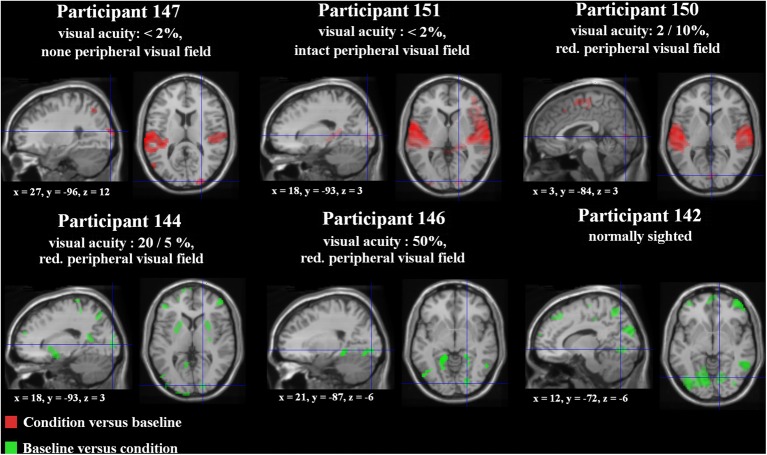
**Special location of the right-hemispheric visual cluster (fixation cross) overlaid on lateral and medial brain structures in each subject (T1 MNI template) based upon the SPM *T*-contrast for forward ultra-fast speech vs. baseline post-training (red = condition vs. baseline, green = baseline vs. condition).** Threshold at *p* < 0.005 uncorrected at voxel level with an extent threshold *k* = 10 (for peak coordinates see Supplementary files 6, 7).

After completion of the training, all subjects displayed significant responses within primary auditory areas of either hemisphere as well as adjacent structures of the superior/middle temporal cortex (Figure 1; Supplementary file 6). Regarding well-trained participants (nos. 147, 150, 144, and 142), visual inspection of the activation clusters within the temporal lobe showed less extension toward the anterior part of the superior temporal gyrus (STG) and sulcus (STS) in subjects with no or low residual vision (nos. 147, 150) as compared to the others (nos. 144, 142). Particularly, the normal-sighted subject (no. 142) displayed strong anterior temporal lobe activation (Figure 1). Furthermore, participants with low residual vision (nos. 147, 151, 150) activated the occipital lobe, e.g., primary and secondary visual areas (BA 17/18) (Figures 1, 2; Supplementary file 6). The FG was found to “light up” in the subject with no residual vision (no. 147), whereas in other participants (nos. 151, 144, 142) BOLD signal changes were restricted to the inferior temporal gyrus (Supplementary file 6). All subjects showed activation within the left-hemispheric inferior frontal gyrus (IFG). Under the applied threshold, left IFG activation occurred sometimes as a secondary peak linked to the temporal cluster (Supplementary file 6). All well-trained subjects displayed hemodynamic responses of left SMA (Figure 1; Supplementary file 6), however, in subject no. 147 SMA activation fell below the applied threshold (*x*, *y*, *z* = −3, 6, 69, *T* = 2.24). One subject with high residual vision (no. 144) showed, obviously, BOLD responses within the left- and right-hemispheric frontal cortex, i.e., precentral gyrus (PrCG), middle frontal gyrus (MFG), orbital gyrus (OrbG), and IFG (Figure 1; Supplementary file 6). Some participants presented with additional activation clusters within the parietal lobe and subcortical areas such as thalamus and caudate nucleus. In all subjects, left and/or right cerebellar activation was noted (Figure 1; Supplementary file 6).

Regarding the SPM *T*-contrast vice versa (baseline vs. ultra-fast speech condition), a widespread pattern concerning the parietal, occipital, and frontal lobe occurred, primarily in subjects with normal or residual vision (nos. 144, 146, 142) and predominantly after the training period (Figures 1, 2; Supplementary file 7; for occipital clusters).

#### Post- minus pre-training regarding the ultra-fast speech condition

Regarding the training effect on hemodynamic activation during ultra-fast speech perception, the six subjects showed different individual response patterns:

Direct comparison of hemodynamic activation after and before the learning procedure (SPM *T*-contrast post- vs. pre-training; Figure 1; Supplementary file 8) revealed only in the sole subject (no. 147) with no residual vision and a high training effect significant BOLD signal changes at the level of the occipital lobe, i.e., right primary and bilateral secondary visual cortex (BA 17/18), and the cerebellum. Additionally, this participant displayed significant hemodynamic responses within the bilateral temporal lobe (STG, middle temporal gyrus = MTG, inferior temporal gyrus = ITG, temporal pole = Tp), and to a lesser extent within the frontal (bilateral PrCG, left IFG) and left parietal lobe (postcentral gyrus = PoCG, supramarginal gyrus = SmG). By contrast, subject no. 151, also afflicted with significantly reduced visual acuity, who, nevertheless, had only moderate training success (36% points), did not show enhanced occipital and cerebellar activation after training. An increase of BOLD responses in this subject occurred predominantly within parietal (left PoCG, bilateral angular gyrus = AG, bilateral inferior parietal lobule = IPL), frontal (left PrCG, left IFG, bilateral MFG), and left temporal (STG, Tp) regions. Subject no. 144—with residual vision—displayed increased hemodynamic responses (from pre- to post-training runs) during the forward ultra-fast condition primarily within right frontal areas (right PrcG, bilateral IFG, bilateral MFG, bilateral SMA, right SFG) and left temporal regions (MTG, STG, Tp). The normal sighted individual (no. 142) showed a positive training effect within bilateral MTG, left PrCG and SMA, bilateral IFG, and left parietal lobe (SmG, IPL). Two further subjects (nos. 150, 146) did not display any significant response differences between pre- and post-training measurements. Most of the subjects (except for subject no. 147) showed a training-induced decrease in activation of temporal regions (MTG, STG; see Figure 1 and Supplementary file 8).

#### Post- minus pre-training regarding the null-event (baseline)

Considering pre- and post-training runs in a concatenated sequence, the subject with no residual vision and a significant training effect (no. 147) showed also a baseline shift (regarding the null-event) in hemodynamic activity between post- and pre-training runs (SPM *T*-contrast, Figure 1; Supplementary file 9 “post- vs. pre-training regarding the null-event (=baseline)”). Thereby, increased activation affected similar regions as described for the forward ultra-fast speech condition (see above paragraph: Post- minus pre-training regarding the ultra-fast speech condition): bilateral occipital cortex and cerebellum, distinct clusters within the bilateral temporal and frontal lobe. In the remaining subjects (nos. 151, 150, 144, 146, 142) an increase of baseline activation was found only within some small clusters: right STG/MTG, PrCG, SmG, IFG, and subcortical regions.

Decreased baseline activity after training was observed in subject no. 151 (weak training effect, low residual vision) within some regions of the occipital lobe whereas the remaining subjects showed such a decrease only in small frontal clusters (Figure 1; Supplementary file 9).

#### Interaction of the experimental conditions (ANOVA)

**Figure 3:**
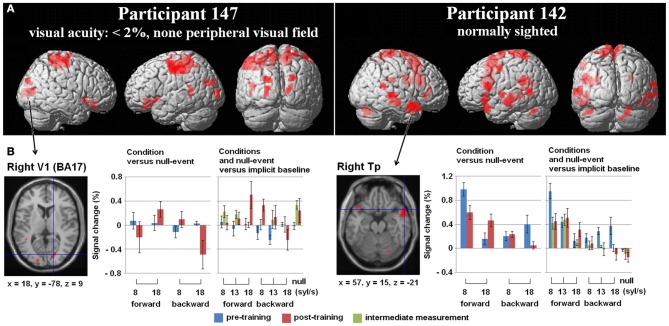
**(A)** Three-Way interaction of an ANOVA (conditions vs. baseline), including the factors *Mode* (forward/backward speech), *Speech rate* (8/18 syl/s), and *Training* (pre-/post-training) exemplified for two single subjects—the one with no residual vision and the normal sighted, both with comparable training success [*p* < 0.005 uncorrected, *k* = 10 voxels]. Variance was calculated across five runs per subject and training stage. **(B)** Hemodynamic effects within one selected cluster from the ANOVA regarding each session (pre-, post-training as well as the intermediate measurement): (i) the right primary visual area (V1) of the blind individual and (ii) the right temporal pole (Tp) of the sighted subject. Values of percent signal change are shown separately for the moderately fast (8 syl/s) and ultra-fast (18 syl/s) condition vs. baseline (left plot) as well as separately for the conditions and the null-event vs. the implicit baseline each (right plot). In the blind subject, right V1 activation during the ultra-fast speech condition increased after the training, whereas the reversed condition showed a decrease (note that also the null-event increased). Since no V1 activation or deactivation was found at pre-training measurements, functional reorganization is suggested. By contrast, since the Tp activation of the sighted person already existed in the pre-training stage during moderately fast speech processing and increased post-training during ultra-fast speech perception, redistribution is assumed to be the neuro-plastic mechanism.

In order to delineate the network used (i) by a participant with no residual vision as well as (ii) a normal-sighted person during processing of ultra-fast speech (18 syl/s) after the training period, the three-way interaction *Mode* × *Speech rate* × *Training* was computed and found to achieve significance in both cases (Figure 3A; Supplementary file 10).

In subject no. 147 (no residual vision), bilateral occipital activation clusters emerged, i.e., left secondary visual areas (BA18) and a right primary visual area (BA17). Further, left temporal (MTG), bilateral fronto-parietal (PrCG, PoCG, paracentral lobule = Pcl), and right frontal (superior frontal gyrus = SFG, OrbG) areas were found to be activated. Considering *post-hoc* hemodynamic effects (values of percentage signal change) of the forward and reversed moderately fast (8 syl/s) and ultra-fast (18 syl/s) conditions (vs. baseline) within the right-hemispheric primary visual area, BOLD response increased after the training (compared with pre-training) during the forward ultra-fast—but not moderately fast—condition, whereas both reversed speech conditions remained at the level of zero or showed deactivation (Figure 3B, left plot).

In the sighted participant (no. 142), bilateral temporal (MTG, Tp), bilateral cerebellar (Cb), left frontal (SMA, IFG, PrCG), and parietal regions (left SmG, right Pcl) showed a significant three-way interaction. *Post-hoc* analysis found increased right temporal pole activation after training during the forward ultra-fast speech condition, but revealed this area also to be activated before training during the moderately fast speech condition. Additionally, this region showed selectivity to forward (compared with reversed) speech (Figure 3B, left plot).

Regarding the training-induced baseline shift (null-event; Figure 3B, right plot), hemodynamic activity during the null-event was stronger after the training period in the blind individual whereas the normal-sighted participant showed an opposite tendency. The baseline shift in the blind subject was even stronger during the intermediate measurement at a training stage of 13 syl/s (not included in the ANOVA).

### Regions-of-interest (ROI) analyses

#### Right-hemispheric visual cortex

**Figure 4:**
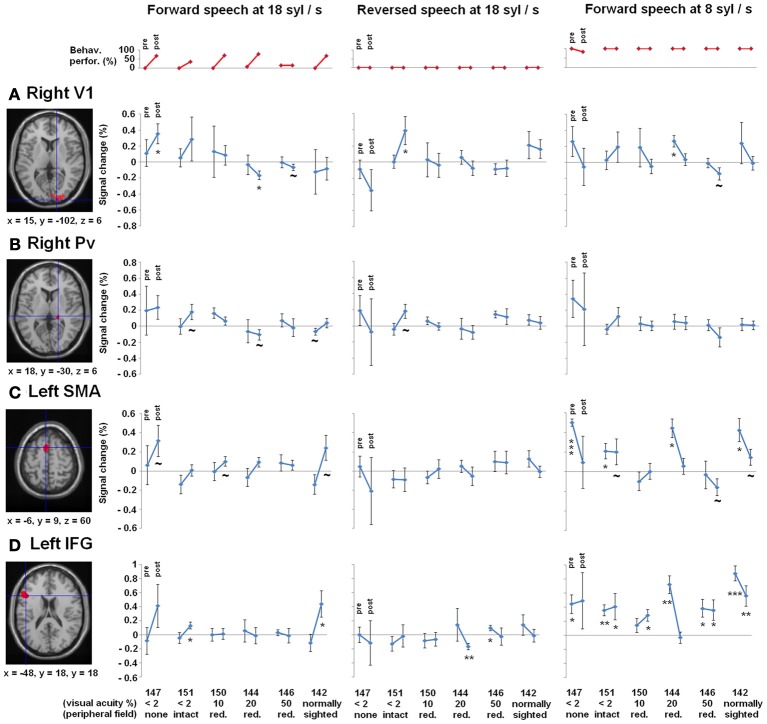
**Regions-of-interest (ROI) analyses, (A) right primary visual area (V1) and (B) right pulvinar (Pv), (C) left supplementary motor area (SMA) as well as the (D) left inferior frontal gyrus (IFG)—all clusters derived from Dietrich et al. (2013), overlaid on an anatomical T1 template.** Upper plot (red): Behavioral performance of understanding ultra-fast speech (18 syl/s) pre- and post-training. Participants are arranged according to their visual acuity. Lower plots (blue): Percent signal change during forward (left) and reversed (middle) ultra-fast speech (18 syl/s) as well as forward moderately fast (8 syl/s) speech (right) pre- and post-training (vs. baseline). Significant differences from zero (one sample *T*-tests): ^*^p < 0.05, ^**^p < 0.01, ^***^p < 0.001, ~ *p* < 0.1.

Concerning right V1 (Figure 4A), the tests of percent signal changes addressed the difference of the values from zero and the differences between conditions. Only subject no. 147 (no residual vision) showed significantly positive values under the forward ultra-fast speech condition following the training sessions (*T* = 2.870, *p* < 0.05) and, additionally, higher values under the forward as compared to the reversed ultra-fast speech condition post-training (*T* = 2.479, *p* < 0.05). Subject no. 151 (low residual vision) achieved positive values under the reversed ultra-fast condition post-training (*T* = 2.248, *p* < 0.05), whereby forward ultra-fast speech not did yield any significant differences as compared to reversed utterances. Subject no. 150 (low residual vision) displayed neither significant positive/negative values nor significant differences between forward and reversed speech under the ultra-fast speech condition after the training sessions. Subjects nos. 144 (*T* = −3.123, *p* < 0.05), 146 (*T* = −1.637, *p* = 0.09), and 142 (*T* = −0.598, *p* = 0.291) showed negative values or did not differ from zero during the forward ultra-fast speech condition post-training. Furthermore, these latter subjects did not show any differences between forward and reversed ultra-fast speech post-training. In addition, significantly positive values under the moderately fast speech condition pre-training (*T* = 3.735, *p* <.05) and selectivity to forward compared with reversed speech (*T* = 1.944, *p* < 0.05) could be observed exclusively in subject no. 144.

**Figure 5:**
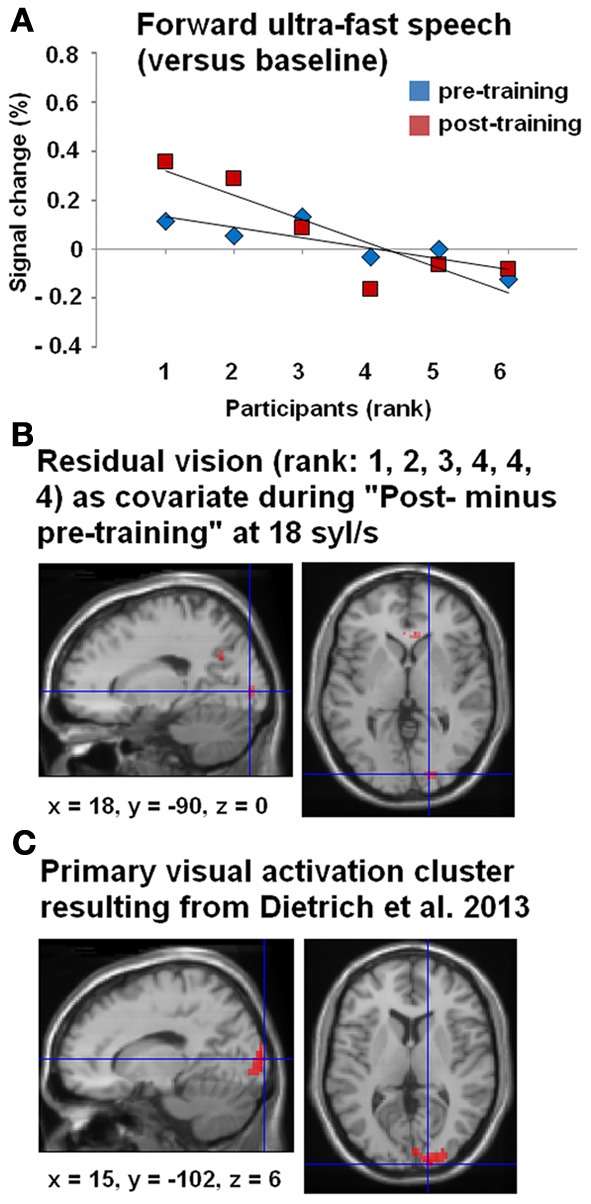
**(A)** Correlations between BOLD responses during the forward ultra-fast (18 syl/s) speech condition (vs. baseline) and the participants, arranged according to their residual vision (given as ranks 1–6: 1 = no residual vision and 6 = normally sighted within the right-hemispheric primary visual activation cluster, drawn from Dietrich et al. (2013) (see panel **C**). Values of percent signal changes refer to the pre- (blue) and post-training (red) measurements of each subject. **(B)** Whole-brain covariance analysis (SPM *T*-contrast “post- minus pre-training,” forward ultra-fast speech condition) with residual vision as a non-linear covariate (rank 1, 2, 3, 4, 4, 4)—hypothesizing that primary visual activation will only change in subjects with no or very low residual vision. The location of this effect in visual cortex was similar to the activation cluster observed in Dietrich et al. (2013) **(C)**.

Furthermore, a significant negative correlation between residual vision and the percent signal change during the forward ultra-fast speech condition (vs. baseline) could be detected before (ρ = −0.77, *p* < 0.05) and after the training sessions (ρ = −0.83, *p* < 0.05), with a tendency toward a steeper regression line at the post-training examination (*T* = −1.569, *p* < 0.1) (Figure 5A). By contrast, no significant correlations emerged during application of moderately fast speech or during the reversed ultra-fast speech condition—neither at pre- nor post-training measurements.

All three subjects with no/low residual vision (rank 1, 2, 3) showed positive values of percent signal change compared to subjects with residual vision (rank 4, 5, 6) with respect to the post- and pre-training stages (Figure 5A). Therefore, a whole-brain covariance analysis was performed with the residual vision as non-linear covariate (rank 1, 2, 3, 4, 4, 4), based on the hypothesis that training-induced enhancement of primary visual activation occurs only in subjects with no or low residual vision. Indeed, the training effect for forward ultra-fast speech (SPM *T*-contrast post- vs. pre-training) was associated with hemodynamic responses of right V1 (Figure 5B) at a similar location as the activation cluster observed in our previous group study (Dietrich et al., 2013; Figure 5C).

#### Right-hemispheric pulvinar

As concerns right Pv, neither pre- nor post-training fMRI evaluation yielded any percent signal change values which significantly differed from zero (Figure 4B). Furthermore, no significant differences emerged between forward and reversed ultra-fast speech conditions post-training (Figure 4B). However, across the entire group of participants, a significant negative correlation between residual vision and percent signal change during forward ultra-fast speech was found at post-training measurements (ρ = −0.77, *p* < 0.05) in right Pv. Prior to the learning procedure, the correlation also showed a negative trend (ρ = −0.71, *p* = 0.055).

#### Left-hemispheric SMA and IFG

As concerns left-hemispheric SMA, all subjects with training effects (= all except no. 146) showed a positive change in percent signal change across follow-up under the forward ultra-fast speech condition (Figure 4C, left plot). By contrast, negative changes from the pre- to the post-training values of this measure could be observed at the level of SMA under the forward moderately fast condition across most subjects (descriptive; Figure 4C, right plot). Considering left IFG, significant positive values of percent signal change were found during the forward moderately fast speech condition across most subjects pre- as well as post-training (Figure 4D). By contrast, only two subjects (nos. 151, 142) showed significant positive values of percent signal change during the forward ultra-fast speech condition post-training, whereas during the backward ultra-fast speech condition negative values could be observed within one subject (no. 144) (Figure 4D).

**Figure 6:**
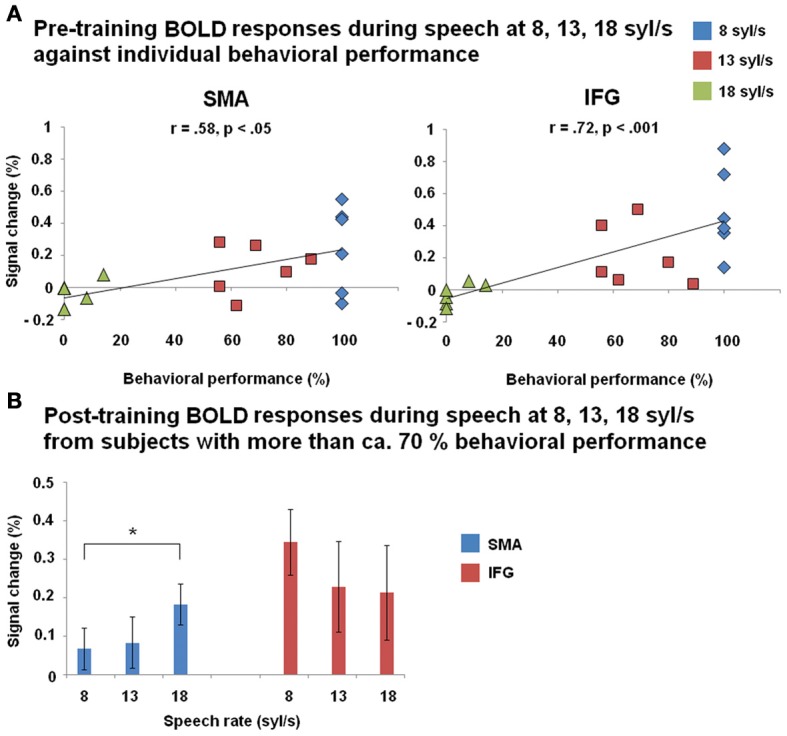
**(A)** Correlation between BOLD responses during forward speech pre-training and individual behavioral performance within left SMA and inferior frontal gyrus (IFG) across all three speech rates (8, 13, 18 syl/s) and across all subjects. **(B)** BOLD responses during forward speech post-training for each speech rate (8, 13, 18 syl/s) within left SMA and IFG. Only subjects with a performance level of more than ca. 70% at speech rate (8 syl/s: *n* = 6; 13 syl/s: *n* = 6; 18 syl/s: *n* = 4) are included. Significant differences (paired *T*-test) emerged between moderately fast and ultra-fast speech within left SMA.

Before training, a significant positive correlation between BOLD responses and behavioral performance (Figure 6A) was found across all three speech rates (8, 13, 18 syl/s) of the forward speech conditions and across all subjects within left SMA (*r* = 0.58, *p* < 0.05) as well as IFG (*r* = 0.72, *p* < 0.001). After training, most subjects (except of nos. 151 and 146 with respect to the ultra-fast speech condition) performed all speech rates (8, 13, 18 syl/s) up to a level of at least ca. 70% correctly reproduced syllables. However, post-training BOLD responses of these subjects with at least ca. 70% performance during the ultra-fast speech condition (vs. baseline, *n* = 4) were significantly higher than during moderately fast speech (*n* = 6) within SMA (*T* = −4.009, *p* < 0.05), but not within IFG (*T* = 1.639, *p* = 0.162) (Figure 6B). Expectedly, against the background that four of the six subjects reached comparable training effects, their residual vision did not significantly correlate with BOLD responses in left SMA or IFG neither pre- nor post-training (*p* > 0.1 for all tests).

## Discussion

### Behavioral training effects

Four of the six participants showed—independently of their residual visual functions—a significant improvement of ultra-fast (18 syl/s) speech comprehension, in terms of a performance level of ca. 70% points after daily training sessions. And the three vision-impaired subjects of this subgroup reported to benefit during daily life from their ability to use screen-reading text-to-speech devices. By contrast, poor (36% points) or no training effects could be observed in the two remaining individuals. So far, none of the participants achieved the pre-specified target of more than 80 % correctly repeated words at a speech rate of 18 syl/s. Since the participants underwent fMRI measurements as soon as they themselves had the impression to meet this criterion, they lacked an external control over actual performance. The sighted control subject of the present study reported that she had “reached her limits” and that she feels additional training would not yield further improvements of ultra-fast speech perception. By contrast, a further extension of the training period (so far ca. 6 months) might have been beneficial in case of vision loss, since higher performance levels have been documented in other blind individuals (Moos and Trouvain, 2007). Most noteworthy, residual vision in terms of visual acuity in the four subjects with a significant—and similar—increase of post-training behavioral performance ranged from none to 100%. Thus, this clinical parameter, obviously, did not constrain the acquisition of ultra-fast speech perception capabilities. It cannot be excluded, however, whether and in how far subjects with low residual vision might be able to further enhance ultra-fast speech comprehension in case of more expanded training procedures.

### Post-training brain networks of ultra-fast speech comprehension (whole-brain analyses)

#### Common activation “nodes” across subjects

Clinical and functional imaging data indicate a contribution of left IFG to speech perception—at least in case more demanding segmentation processes and/or working memory operations are involved (Burton et al., 2000). Furthermore, hemodynamic activation of language processing areas such as IFG has been assumed to represent an index of actual speech comprehension (Poldrack et al., 2001). The present study revealed post-training left-hemispheric IFG responses across all subjects, indicating intelligibility of ultra-fast spoken language even if the training effect was low (subject no. 146 showed no training effects, nevertheless, performance level at baseline had amounted to 14%). Furthermore, left-hemispheric PrCG and the cerebellum displayed significant BOLD signal changes during forward ultra-fast speech post-training. These observations are in line with clinical and functional imaging data pointing at a contribution of those structures—under specific circumstances—to auditory speech perception. For example, the cerebellum has been found engaged in the encoding of specific temporal-linguistic information during word identification tasks (Mathiak et al., 2002).

#### Descriptive analyses of individual response patterns

Four participants showed a significant—and similar—increase of post-training behavioral performance (ca. 70% points), independent of residual vision capabilities (<2, 10, 20, 100% visual acuity). Most noteworthy, different individual hemodynamic response patterns could be observed in these cases, indicating processing strategies of ultra-fast speech understanding to vary across these subjects.

Training of ultra-fast speech comprehension was found to induce hemodynamic activation at the level of the occipital lobe in three subjects with no or low residual vision. The strongest effects (also significant in the post- minus pre-training comparison) emerged in subject no. 147, a participant lacking any residual visual functions, and such responses to accelerated spoken language could not be observed at the baseline examination. These findings provide tentative support for a relationship between ultra-fast speech perception and right-hemispheric V1 activation—in case the central-visual system is deprived of modality-specific afferent input. Based on our previous studies, it has been suggested that collaboration of right auditory cortex and ipsilateral primary visual area allows for the implementation of a signal-driven timing mechanism related to syllabic modulation of the speech signal (Dietrich et al., 2013; Hertrich et al., 2013a,b). More specifically, subjects able to recruit the visual cortex during spoken language comprehension appear to install an additional information channel conveying temporal cues on syllable structure to the frontal lobe which then help to trigger phonological encoding processes and, as a consequence, to support verbal working memory operations (Hertrich et al., 2013b). Since the visual system seems to compensate for the temporal limitations of the auditory system under these conditions, this strategy might allow for a further increase in ultra-fast speech comprehension capabilities beyond the level that was achieved in the present training study.

Besides increased V1 activation during the ultra-fast speech condition, subject 147 also showed significant changes in the null-event if the runs across the pre- and post-training sessions were concatenated for analysis. Interestingly, this increase was strongest in the session at an intermediate training level when the ultra-fast condition did not yet activate right V1 (vs. baseline). This “baseline shift” might result from actual activity during the null event. However, it could also reflect a discrepancy between the explicit (null-event) and the implicit baseline, which might indicate that the BOLD response in V1 is not strictly aligned with the stimuli across training stages. This inconsistency might be bound to the emergence of reorganization processes—rather than just redistribution of brain activity as reported by Kelly and Garavan (2005)–since occipital cortex was not active prior to the pre-training session and since a new area has been recruited during the learning procedure.

Interestingly, subject no. 151 (with low visual acuity similar to subject no. 147) showed a strong reduction of the null-event activity within the occipital lobe at the post- as compared to the pre-training session, but no increase of activation during the ultra-fast speech condition. The low training effect of this subject seems to be in line with the absence of significant V1 activation during the ultra-fast condition. However, the strong changes of the null-event activity might indicate that also in this subject training-induced reorganization is possible.

By contrast, subject no. 150 (10% visual acuity) did not show any training-induced changes in occipital cortex activity and, thus, visual system recruitment for the sake of ultra-fast speech perception seems to be impossible.

It should be noted that the post-training hemodynamic response pattern of subject no. 147, displaying the strongest response of right visual cortex, encroaches, in addition, upon left-hemispheric primary (BA17) and bilateral secondary (SOG, MOG) visual areas. As an explanation, the recruitment of occipital areas at either side of the brain might reflect central-visual support of several linguistic functions during ultra-fast speech comprehension, e.g., both segmental (left hemisphere) and suprasegmental/prosodic (right hemisphere) processing. Against the background of previous findings (Dietrich et al., 2013; Hertrich et al., 2013b), strong right-hemispheric lateralization of the primary visual area seems to be associated with a prosodic function, i.e., triggering syllable onsets within a metric pattern. Thus, a somehow unfocused reorganization pattern might be further stabilized after getting more experienced with ultra-fast speech materials. Continuation of the training procedure at high rates could result, conceivably, in a less distributed occipital activation pattern centered around the right primary visual area.

In line with our preceding single-case and group studies on ultra-fast speech perception (Hertrich et al., 2009; Dietrich et al., 2013), post-training fMRI revealed hemodynamic activation of left FG in response to ultra-fast speech materials in subject no. 147, characterized by the strongest engagement of right-hemisphere visual cortex. FG is embedded into the so-called ventral route of the central-visual system which, especially, supports object recognition (e.g., Haxby et al., 1991), but may contribute to phonological operations as well (e.g., Cone et al., 2008). Thus, left FG as a secondary phonological area might expand the phonological network and help to cope with the higher processing demands during ultra-fast speech perception.

Presumably, right-hemispheric frontal lobe activation, including PrCG, MFG, OrbG, and IFG as observed in subject no. 144, might reflect a network associated with phonological word stress processing during ultra-fast speech comprehension. In previous studies, neural correlates of word stress as compared to vowel quality encoding (Klein et al., 2011) or auditory processing of bi-syllabic pseudo-words (Tervaniemi et al., 2009) were found within a widespread fronto-temporal activation pattern including, among others, right-hemispheric activation clusters resembling the response pattern of subject no. 144. Against this background, it may be assumed that a prosodic/metric pattern was generated, predicting continuous word stress or metrical stress sequences during listening to ultra-fast speech. Such a focus on stressed syllables—ignoring more or less the unstressed ones—might help to overcome the backward masking effects of high speaking rates. However, this strategy of ultra-fast speech comprehension, conceivably, poses higher demands on the auditory system with regard to the processing of word stress information, interpolation of unstressed syllables, and the mapping of these patterns onto articulatory motor plans. This assumption is in line with reports of increased activation of the auditory association cortices and left ventral premotor cortex after training to understand time-compressed speech in sighted individuals (Adank and Devlin, 2010). Although such a compensation strategy based upon prosodic/metric redundancies of verbal utterances might facilitate ultra-fast speech comprehension to a certain extent, it may not be able to really enhance the temporal resolution at the level of the syllable and, therefore, might require increased top-down effort of language processing.

Distinct hemodynamic activation of the temporal pole at either side was found after training of ultra-fast speech comprehension in the sighted subject no. 142. Combinatorial processes at sentence-level (Hickok and Poeppel, 2007) and the evaluation of affective semantics (Ethofer et al., 2006) have been associated with hemodynamic responses of the anterior temporal lobe, indicating holistic rather than compositional (i.e., hierarchical analysis of segments, syllables, and morphemes, and phrases) semantic processing under these conditions. Such top-down mechanisms—based upon contextual/semantic pre-information—may facilitate sound-to-semantic mapping processes even in case of incomplete phonological decoding. Although the use of this strategy should overcome to a certain extent the backward masking effects of ultra-fast speech signals, we may speculate that subjects applying this strategy will have difficulty understanding the logical structure of more complex sentences. Moreover, the present (sighted) subject who might have used this strategy did not recruit any new area as in the case of the blind participant. Instead, activity existing pre-training during the moderately fast speech condition was found increased post-training during the ultra-fast speech condition. Thus, redistribution of activity as defined in Kelly and Garavan (2005) is assumed to be the underlying mechanism for plasticity.

Summarizing these descriptive analyses of distinct individual activation patterns, subjects appear to use different strategies during ultra-fast speech perception depending, among other things, on residual visual functions. In case of very low residual vision, the visual system seems to be recruited as a means to enhance actual temporal resolution of the acoustic signal via implementation of an extra-channel conveying information on syllabic structure to verbal working memory buffers. This new strategy concomitant with the recruitment of an additional area can be considered a variant of functional reorganization. If the visual system is not at a subject's disposal for spoken language comprehension, listeners may rely on already available semantic top-down strategies facilitating lexical access, based upon, e.g., redundant aspects of verbal materials. Under these conditions, a stronger engagement of (some components of) the classical language network might help to speed up the encoding processes, providing an example of neuroplasticity due to redistribution of brain activity.

### The impact of residual vision and behavioral performance on the responses of the visual system (ROI analyses)

During forward ultra-fast speech perception, residual vision was found to have a significant impact on the activation of the right-hemispheric primary visual cortex (V1) and ipsilateral pulvinar (Pv), a subcortical structure projecting to the occipital lobes. More specifically, a negative correlation emerged in terms of decreased hemodynamic activation concomitant with increased residual vision. The respective regression line showed a trend toward a steeper decline at the post-training measurements, indicating a differential impact of residual vision on the training effects. Thus, residual visual capacities appear to constrain the resources of audiovisual reorganization during ultra-fast speech perception. The critical threshold seems to be centered around acuity values of 10 to 20%, whereas a more efficient use of the visual cortex such as in subject no. 147 even may require residual vision to fall below a level of only 2%.

A previous study investigating cross-modal reorganization in a vision-impaired subject who had suffered from severe visual acuity reduction with no evidence of visual field loss, reported populations of visual neurons not critical for a subject's remaining low vision to be recruited for tactile information processing (Cheung et al., 2009). The observation of such a retinotopy-related functional segregation of neurons bound to residual vision, on the one hand, and cross-modal processing, on the other, might explain why audiovisual plasticity did not occur in the subjects of our group with normal or high residual vision. Furthermore, visual acuity rather than the peripheral visual field seems to be the crucial prerequisite of a recruitment of the visual system (Cheung et al., 2009). The present data confirm this suggestion since, e.g., subject no. 151 with no visual acuity, but intact peripheral visual fields showed increased hemodynamic activation of the visual system at post-training measurements although the subject showed a moderate training effect only. By contrast, participant no. 144 suffering from a strongly reduced visual field did not activate right visual cortex after the training sessions during the forward ultra-fast speech condition, presumably, because visual acuity was not markedly reduced. Consequently, the foveal representation within the visual system seems to be the target of cross-modal reorganization processes during the acquisition of ultra-fast speech comprehension. In line with this hypothesis, training of ultra-fast speech comprehension induced strong visual cortex activation in a subject with no residual vision (no. 147). In spite of good training effects, only low hemodynamic responses within the visual system could be observed in the subject suffering from residual 10% visual acuity (no. 150).

Interestingly, forward moderately fast speech did not induce any distinct hemodynamic activation of visual cortex after training. It can be assumed, thus, that the speech task has to be sufficiently challenging in order to induce significant activation in the visual system. In fact, passive listening to sounds does not give rise to occipital activation in blind subjects (Arno et al., 2001). Accordingly, visual areas in the blind were not activated by the mere presence of sound, but were involved in attentive perception of changes in the auditory environment (Kujala et al., 2005). Attention is suggested to play an important role during ultra-fast speech comprehension as well—in terms of the detection of almost entirely masked syllable onsets (Dietrich et al., 2013; Hertrich et al., 2013b). However, significant visual cortex activation was also found at pre-training examination during the moderately fast speech condition selectively to forward (compared to reversed) speech in a subject with high residual vision (no. 144). As an explanation, hemodynamic responses of visual cortex during speech perception might reflect actual visual imagery. The subjects' high familiarity with speech signals in association with speaking faces during language acquisition might facilitate such effects also in late-blind subjects. In a previous study, addressing the reverse relationship of the two sensory modalities, perception of visual lip movements during silent syllable production was found to evoke primary auditory BOLD responses, indicating strong audio-visual interactions during speech perception (Hertrich et al., 2010).

Taken together, the highest activation levels of the visual system were found in case of (i) absent residual vision and (ii) good training effects. Only subject no. 147 met these two criteria while the remaining subjects fell short to either one or both characteristics. As concerns the latter participant, visual system recruitment was selectively limited to forward—and, thus, principally intelligible—utterances. As a rule, right Pv displayed a profile of BOLD responses similar to right visual cortex. Again, these findings point at a contribution of this subcortical structure to ultra-fast speech perception. Conceivably, the observed interactions of Pv and visual cortex reflect the operation of an audiovisual interface in that the secondary visual pathway provides the central-visual system with auditory input (see Hertrich et al., 2013b, for more details).

### Vision-independent and performance-dependent changes of hemodynamic activation of left SMA and IFG (ROI analysis)

Pre-training BOLD responses within left IFG and SMA were found to be significantly correlated—independently from residual vision—with the percentage of correctly understood speech materials, pooled across speech rates (8, 13, 18 syl/s). Consequently, both left IFG and SMA activation are associated with speech comprehension. More specifically, it is suggested that left IFG is involved in the speech production-related phonological reconstruction of perceived speech material (Burton et al., 2000). However, not all participants with significant training effect showed significant left IFG activation with respect to the cluster resulting from Dietrich et al. (2013) after the training period. Therefore, some subjects seem not to reach the threshold with respect to the relatively large cluster resulted from (Dietrich et al., 2013). However, the whole-brain analyses revealed post-training left-hemispheric IFG activation in subjects with significant training effect.

Prior to training, hemodynamic activation within left SMA during the “moderately fast condition” showed considerable variation across individuals. Recruitment of left SMA, thus, does not seem to be a prerequisite to speech comprehension *per se* as such. However, all subjects with training effects showed a positive change in percent signal change across follow-up under the forward ultra-fast speech condition. Moreover, left SMA, but not IFG activation post-training was significantly higher during the ultra-fast than the moderately fast speech condition in well-performing subjects. Neither the pre- nor post-training responses of SMA and IFG depended on residual vision, an observation supporting the view of a supra-modal function of these frontal structures, irrespective of input modality. Several studies indicate SMA to engage in the syllabic organization of verbal utterances during speech production (Ziegler et al., 1997; Riecker et al., 2005; Brendel et al., 2010). On a broader scale, this region appears to support timing processes across various sensorimotor and cognitive domains (Rubia and Smith, 2004; Paz et al., 2005). Furthermore, clinical as well as experimental data point at a contribution of SMA to spoken language perception and verbal working memory operations as well (Schirmer et al., 2001; Smith et al., 2003; Schirmer, 2004; Chung et al., 2005; Geiser et al., 2008). Given that SMA might act as a platform for timing operations related to verbal working memory functions, sensory systems might convey temporal information on syllable structure via left SMA into this short-term storage system. More specifically, the joint activation of auditory and visual areas with left SMA could reflect a signal-driven timing mechanism facilitating the transformation of the perceived acoustic signal into an active pre-articulatory phonetic/phonological output structure in our speech generation system under time-critical conditions (see Hertrich et al., 2013b, for more details).

## Conclusions

Several participants were found able to increase ultra-fast speech (18 syl/s) comprehension up to a level of ca. 70% points, irrespective of residual vision, across a training session of about 6 months. However, subjects seem to deploy distinct patterns of neuroplasticity in order to overcome the bottleneck of temporal resolution associated with high speaking rates: In case of very low residual vision, the right-hemispheric visual system (V1, Pv) was, apparently, recruited indicating functional reorganization at the level of the occipital lobes. By contrast, participants with normal or high residual vision did not show any training-induced responses of the central-visual system. However, they displayed increased activation (redistribution) of several parts of the classical speech processing network. Although both patterns of neuroplasticity resulted in comparable training effects, the strategy of processing ultra-fast speech might significantly differ: In case of a deployment of the visual system, actual temporal resolution of the acoustic signal might increase via the representation of a syllabic trigger cues. If, however, the visual system is not at a subject's disposal for spoken language processing, they may use, as an alternative, a strategy based on informational redundancies of the speech signal. Independent of residual vision, left-hemispheric SMA and IFG activation emerged as a common indicator of speech comprehension. In case of proficient ultra-fast speech comprehension, however, only SMA showed stronger activation during application of ultra-fast as compared to moderately fast test materials, pointing at a critical contribution to spoken language understanding under time-critical conditions.

## Authors contribution

Hermann Ackermann, Ingo Hertrich, and Susanne Dietrich delineated the rationale and developed the design of the study. IH and SD were engaged in data collection and development of analyses methods. SD performed the behavioral and fMRI data analyses, and drafted the first version of the paper. All authors contributed to the final version of the manuscript and approved its content.

### Conflict of interest statement

The authors declare that the research was conducted in the absence of any commercial or financial relationships that could be construed as a potential conflict of interest.

## Supplementary material

Supplementary file 1 **An example for forward moderately fast speech (8 syl/s).** “Wegen den anstehenden wichtigen Prüfungen muss er viel lernen.”

Supplementary file 2 **An example for forward ultra-fast speech (18 syl/s).** “Die Mitfahrerin stellte sich als gute Gesprächspartnerin heraus.”

Supplementary file 3 **An example for reversed moderately fast speech (8 syl/s). See Supplementary file 1**.

Supplementary file 4 **An example for reversed ultra-fast speech (18 syl/s). See Supplementary file 2**.

Supplementary file 5 **An example of the repetition task for ultra-fast speech of blind listeners with ca. 70% comprehension capability.** “Die Karten für das Konzert nächstes Jahr sind bereits jetzt ausverkauft.”

Supplementary file 6 **Coordinates describing the SPM *T*-contrasts “forward ultra-fast speech condition versus baseline” pre- and post-training separately**.

Supplementary file 7 **Coordinates describing the SPM *T*-contrasts “baseline versus forward ultra-fast speech condition” pre- and post-training separately**.

Supplementary file 8 **Coordinates describing the SPM *T*-contrasts “post- versus pre-training” during the forward ultra-fast speech condition (and vice versa)**.

Supplementary file 9 **Coordinates describing the SPM *T*-contrasts “post- versus pre-training” (and vice versa) during the null-event (=baseline)**.

Supplementary file 10 **Coordinates describing the Three-Way interaction “*Mode* × *Speech rate* × *Training*” resulting from the ANOVA exemplified to subject nos. 147 and 142**.
