# Supplementary material for: Training of ultra-fast speech comprehension induces functional reorganization of the central-visual system in late-blind humans
Source: Front Hum Neurosci. 2013 Oct 23;7:701. doi: 10.3389/fnhum.2013.00701 (PMC3805979; doi:10.3389/fnhum.2013.00701)
Supplement: Supplementary file 1 — An example for forward moderately fast speech (8 syl/s). “Wegen den anstehenden wichtigen Prüfungen muss er viel lernen.” [file Presentation1.ZIP › 64044_Dietrich_Data_Sheet_9.DOCX]

| **Supplementary file 9.** Coordinates of the whole-brain analysis of all six participants arranged according to their residual vision (increasing from left to right). Hemodynamic effects of the SPM *T*-contrast “post- versus pre-training regarding the baseline (= null event)” were threshold at *p* < 0.005 at voxel level uncorrected with contiguous voxels *k* = 10. If the value of the x coordinate was negative (-), the peak occurred within the left hemisphere. Italicized numbers labeled sub-peaks. | | | | | | | | | | | | | | | | | | | | | | | | |
| --- | --- | --- | --- | --- | --- | --- | --- | --- | --- | --- | --- | --- | --- | --- | --- | --- | --- | --- | --- | --- | --- | --- | --- | --- |
|  | **147** | | | | **151** | | | | **150** | | | | **144** | | | | **146** | | | | **142** | | | |
| Region | T | MNI coordinate | | | T | MNI coordinate | | | T | MNI coordinate | | | T | MNI coordinate | | | T | MNI coordinate | | | T | MNI coordinate | | |
|  |  | x | y | z |  | x | y | z |  | x | y | z |  | x | y | z |  | x | y | z |  | x | y | z |
|  | | | | | | | | | | | | | | | | | | | | | | | | |
| **Post- versus pre-training (null-event)** | | | | | | | | | | | | | | | | | | | | | | | | |
| ***Temporal lobe*** | | | | | | | | | | | | | | | | | | | | | | | | |
| MTG | 3.7 | 54 | -51 | 3 |  |  |  |  |  |  |  |  |  |  |  |  |  |  |  |  | 3.1 | 66 | -24 | -3 |
|  | 3.5 | -57 | -51 | 15 |  |  |  |  |  |  |  |  |  |  |  |  |  |  |  |  |  |  |  |  |
| STG | *3.0* | *57* | *-48* | *18* |  |  |  |  |  |  |  |  | 2.8 | 66 | -9 | 9 |  |  |  |  |  |  |  |  |
| ITG | 3.6 | -45 | -27 | -24 |  |  |  |  |  |  |  |  |  |  |  |  |  |  |  |  |  |  |  |  |
|  | 3.1 | -60 | -33 | -21 |  |  |  |  |  |  |  |  |  |  |  |  |  |  |  |  |  |  |  |  |
|  | 3.1 | 54 | -30 | -24 |  |  |  |  |  |  |  |  |  |  |  |  |  |  |  |  |  |  |  |  |
| ***Occipital lobe*** | | | | | | | | | | | | | | | | | | | | | | | | |
| MOG | *4.2* | *-36* | *-81* | *15* |  |  |  |  |  |  |  |  |  |  |  |  |  |  |  |  |  |  |  |  |
| FG | *3.9* | *27* | *-66* | *-15* |  |  |  |  |  |  |  |  |  |  |  |  |  |  |  |  |  |  |  |  |
|  | 2.9 | 30 | -51 | -9 |  |  |  |  |  |  |  |  |  |  |  |  |  |  |  |  |  |  |  |  |
| BA18 | 4.5 | -6 | -75 | 18 |  |  |  |  |  |  |  |  |  |  |  |  |  |  |  |  |  |  |  |  |
|  | *4.1* | *6* | *-81* | *18* |  |  |  |  |  |  |  |  |  |  |  |  |  |  |  |  |  |  |  |  |
| BA17 | 3.3 | 18 | -69 | 3 |  |  |  |  |  |  |  |  |  |  |  |  |  |  |  |  |  |  |  |  |
| Cun | 3.1 | -12 | -75 | 36 |  |  |  |  |  |  |  |  |  |  |  |  |  |  |  |  |  |  |  |  |
| ***Frontal lobe*** | | | | | | | | | | | | | | | | | | | | | | | | |
| RecG | 3.1 | -6 | 30 | -18 |  |  |  |  |  |  |  |  |  |  |  |  |  |  |  |  |  |  |  |  |
| PrCG |  |  |  |  | 3.4 | -54 | 0 | 48 |  |  |  |  |  |  |  |  |  |  |  |  |  |  |  |  |
| IFGorb |  |  |  |  |  |  |  |  |  |  |  |  |  |  |  |  |  |  |  |  | 3.4 | 51 | 51 | -3 |
| ***Parietal lobe*** | | | | | | | | | | | | | | | | | | | | | | | | |
| SumG | 3.0 | -63 | -24 | 21 |  |  |  |  | 3.4 | 60 | -39 | 33 |  |  |  |  |  |  |  |  |  |  |  |  |
| ***Other*** | | | | | | | | | | | | | | | | | | | | | | | | |
| Cb | 5.5 | 45 | -75 | -27 |  |  |  |  |  |  |  |  |  |  |  |  |  |  |  |  |  |  |  |  |
|  | *4.5* | *-21* | *-60* | *-15* |  |  |  |  |  |  |  |  |  |  |  |  |  |  |  |  |  |  |  |  |
|  | 4.2 | 33 | -39 | -39 |  |  |  |  |  |  |  |  |  |  |  |  |  |  |  |  |  |  |  |  |
|  | 4.1 | -30 | -39 | -39 |  |  |  |  |  |  |  |  |  |  |  |  |  |  |  |  |  |  |  |  |
| Hipp | 3.0 | -18 | -30 | -9 |  |  |  |  |  |  |  |  |  |  |  |  |  |  |  |  |  |  |  |  |
| Amg | 3.2 | 24 | 0 | -15 |  |  |  |  |  |  |  |  |  |  |  |  |  |  |  |  |  |  |  |  |
| Pal |  |  |  |  |  |  |  |  |  |  |  |  | 3.2 | 27 | -3 | 0 |  |  |  |  |  |  |  |  |
| Put |  |  |  |  |  |  |  |  |  |  |  |  | 3.0 | -27 | 0 | 0 |  |  |  |  |  |  |  |  |
|  |  |  |  |  |  |  |  |  |  |  |  |  |  |  |  |  |  |  |  |  |  |  |  |  |
| **Pre- minus post-training (null-event)** | | | | | | | | | | | | | | | | | | | | | | | | |
| ***Temporal lobe*** | | | | | | | | | | | | | | | | | | | | | | | | |
| ITG |  |  |  |  | 3.4 | -60 | -60 | -9 |  |  |  |  |  |  |  |  |  |  |  |  |  |  |  |  |
|  |  |  |  |  | 2.9 | -45 | -18 | -21 |  |  |  |  |  |  |  |  |  |  |  |  |  |  |  |  |
| ***Occipital lobe*** | | | | | | | | | | | | | | | | | | | | | | | |  |
| Cun |  |  |  |  | 5.4 | -6 | -87 | 33 |  |  |  |  |  |  |  |  |  |  |  |  |  |  |  |  |
| MOG |  |  |  |  | *4.1* | *-24* | *-96* | *15* |  |  |  |  |  |  |  |  |  |  |  |  |  |  |  |  |
| BA17 |  |  |  |  | *3.8* | *12* | *-102* | *12* |  |  |  |  |  |  |  |  |  |  |  |  |  |  |  |  |
|  |  |  |  |  | *2.9* | *-9* | *-87* | *6* |  |  |  |  |  |  |  |  |  |  |  |  |  |  |  |  |
| BA18 |  |  |  |  | *3.7* | *-3* | *-99* | *21* |  |  |  |  |  |  |  |  |  |  |  |  |  |  |  |  |
| FG |  |  |  |  | 4.0 | -39 | -54 | -9 |  |  |  |  |  |  |  |  |  |  |  |  |  |  |  |  |
|  |  |  |  |  | 2.9 | 24 | -45 | -18 |  |  |  |  |  |  |  |  |  |  |  |  |  |  |  |  |
| IOG |  |  |  |  | 3.6 | 48 | -81 | -3 |  |  |  |  |  |  |  |  |  |  |  |  |  |  |  |  |
|  |  |  |  |  | 3.3 | -51 | -66 | -15 |  |  |  |  |  |  |  |  |  |  |  |  |  |  |  |  |
| ***Frontal lobe*** | | | | | | | | | | | | | | | | | | | | | | | | |
| SorG |  |  |  |  | 3.1 | 30 | 66 | -3 |  |  |  |  |  |  |  |  |  |  |  |  |  |  |  |  |
| BA6 |  |  |  |  | 3.3 | -18 | -21 | 54 |  |  |  |  |  |  |  |  |  |  |  |  |  |  |  |  |
| MorG |  |  |  |  | 2.9 | -30 | 60 | -12 |  |  |  |  |  |  |  |  |  |  |  |  |  |  |  |  |
| PrCG |  |  |  |  |  |  |  |  | 3.0 | -60 | 3 | 27 |  |  |  |  |  |  |  |  | 3.4 | 42 | -15 | 66 |
|  |  |  |  |  |  |  |  |  |  |  |  |  |  |  |  |  |  |  |  |  | 2.9 | -36 | 0 | 57 |
| PoCG |  |  |  |  |  |  |  |  | *2.8* | *-54* | *-3* | *39* |  |  |  |  |  |  |  |  | 3.8 | 66 | -9 | 33 |
| MFG |  |  |  |  |  |  |  |  |  |  |  |  | 3.1 | 39 | 36 | 42 |  |  |  |  |  |  |  |  |
| ***Parietal lobe*** | | | | | | | | | | | | | | | | | | | | | | | | |
| AG |  |  |  |  |  |  |  |  |  |  |  |  | 3.1 | 51 | -72 | 30 |  |  |  |  |  |  |  |  |
| SPL |  |  |  |  | 3.0 | 30 | -75 | 54 |  |  |  |  |  |  |  |  |  |  |  |  |  |  |  |  |
| Prc |  |  |  |  | 4.5 | 3 | -60 | 63 |  |  |  |  |  |  |  |  |  |  |  |  | 3.2 | -6 | -45 | 42 |
|  |  |  |  |  | 3.3 | 12 | -39 | 6 |  |  |  |  |  |  |  |  |  |  |  |  |  |  |  |  |
|  |  |  |  |  | 3.1 | 3 | -42 | 57 |  |  |  |  |  |  |  |  |  |  |  |  |  |  |  |  |
| ***Other*** | | | | | | | | | | | | | | | | | | | | | | | | |
| Cb |  |  |  |  | 3.8 | -6 | -72 | -21 |  |  |  |  |  |  |  |  |  |  |  |  |  |  |  |  |
|  |  |  |  |  | *3.3* | *12* | *-69* | *-39* |  |  |  |  |  |  |  |  |  |  |  |  |  |  |  |  |
|  |  |  |  |  | *3.5* | *-33* | *-60* | *-42* |  |  |  |  |  |  |  |  |  |  |  |  |  |  |  |  |
| Amg |  |  |  |  | 3.1 | 33 | -3 | -15 |  |  |  |  |  |  |  |  |  |  |  |  |  |  |  |  |
|  |  |  |  |  | 3.1 | -21 | -3 | -12 |  |  |  |  |  |  |  |  |  |  |  |  |  |  |  |  |
| CC |  |  |  |  | 3.1 | 6 | -45 | 30 |  |  |  |  |  |  |  |  |  |  |  |  |  |  |  |  |
|  |  |  |  |  |  |  |  |  |  |  |  |  |  |  |  |  |  |  |  |  |  |  |  |  |
| Abbreviations: AG, angular gyrus; Amg, amygdala; BA, Brodman area; Cb, cerebellum; CC, cingulate cortex; Cun, cuneus; FG, fusiform gyrus; Hipp, hippocampus; IFG, inferior frontal gyrus; IFGorb, inferior frontal gyrus pars orbitalis; IOG, inferior occipital gyrus; ITG, inferior temporal gyrus; MorG, middle orbital gyrus; MTG, middle temporal gyrus; Pal, pallidum; PoCG, postcentral gyrus; Prc, precuneus; PrCG, precentral gyrus; Put, putamen; MFG, middle frontal gyrus; MOG, middle occipital gyrus; MTG, middle temporal gyrus; RecG, rectal gyrus; SFG, superior frontal gyrus; SMA, supplementary motor area; SmG, supramarginal gyrus; SOG, superior occipital gyrus; SorG, superior orbital gyrus; SPL, superior parietal lobule; STG, superior temporal gyrus. | | | | | | | | | | | | | | | | | | | | | | | | |
