# Supplementary material for: Training of ultra-fast speech comprehension induces functional reorganization of the central-visual system in late-blind humans
Source: Front Hum Neurosci. 2013 Oct 23;7:701. doi: 10.3389/fnhum.2013.00701 (PMC3805979; doi:10.3389/fnhum.2013.00701)
Supplement: Supplementary file 1 — An example for forward moderately fast speech (8 syl/s). “Wegen den anstehenden wichtigen Prüfungen muss er viel lernen.” [file Presentation1.ZIP › 64044_Dietrich_Data_Sheet_10.DOCX]

| **Supplementary file 10.** Coordinates of the whole-brain ANOVA analysis of two participants (a) normal sighted (no. 142) and (b) without residual vision (no. 147) both with a significant training effect (ca. 70 %). Hemodynamic effects of the three-way interaction *Mode* × *Speech rate* × *Training* were threshold at *p* < 0.005 at voxel level uncorrected with contiguous voxels *k* = 10. Italicized numbers labeled sub-peaks. | | | | | | | | | | | | | |
| --- | --- | --- | --- | --- | --- | --- | --- | --- | --- | --- | --- | --- | --- |
| Region | Side | Cluster size (voxel) | T | MNI  coordinate | | | Region | Side | Cluster size (voxel) | T | MNI  coordinate | | |
|  |  |  |  | x | y | z |  |  |  |  | x | y | z |
| **a) Participant no. 142 (normal sighted)** | | | | | | | **b) Participant no. 147 (no residual vision)** | | | | | | |
| SMA, BA6 | left | 395 | 21.5 | -3 | 9 | 54 | PrCG, BA6 | left | 294 | 14.6 | -39 | -9 | 63 |
| MTG | left | 146 | 19.4 | -51 | -21 | -18 | PoCG |  |  | *13.1* | *-30* | *-33* | *54* |
| Tp | left | 87 | 13.8 | -48 | 18 | -24 | IPL |  |  | *12.8* | *-30* | *-57* | *54* |
| IFG | left |  | *10.9* | *-36* | *24* | *-9* | Pcl | left | 120 | 16.8 | -9 | -36 | 63 |
| SMA | left | 79 | 10.3 | -9 | -3 | 75 | SPL |  |  | *13.7* | *-18* | *-45* | *66* |
| SmG | left | 77 | 17.0 | -48 | -48 | 24 | MCC |  |  | *10.7* | *-15* | *-36* | *45* |
| IFG | left | 77 | 17.3 | -54 | 18 | 15 | RecG | left | 62 | 16.7 | -9 | 33 | -15 |
| MTG | left | 38 | 16.7 | -57 | -57 | 6 | SOG | left | 44 | 13.0 | -15 | -84 | 24 |
| PrCG | left | 27 | 12.5 | -54 | 0 | 48 | CN | left | 26 | 16.4 | -3 | 12 | -6 |
| Cb | left | 21 | 13.4 | -42 | -63 | -30 | MOG, BA18 | left | 23 | 11.3 | -18 | -93 | 6 |
| Pal | left | 13 | 14.1 | -18 | -6 | -3 | MTG | left | 19 | 11.1 | -42 | -48 | 9 |
| Pcl | right | 156 | 15.2 | 3 | -33 | 72 | STG |  |  | *11.0* | *-45* | *-42* | *18* |
| SPL | left |  | *12.9* | *-18* | *-48* | *69* | PoCG | right | 165 | 17.2 | 18 | -39 | 63 |
| MTG | right | 133 | 14.7 | 51 | -21 | -15 | Pcl |  |  | *12.3* | *6* | *-27* | *60* |
| STG | right |  | *13.9* | *66* | *-27* | *3* | SMA, BA6 |  |  | *12.3* | *6* | *-21* | *63* |
| Tp | right | 117 | 22.6 | 57 | 15 | -21 | PrCG |  |  | *12.2* | *27* | *-30* | *66* |
| NC | right | 55 | 14.7 | 18 | 0 | 24 | PrCG | right | 34 | 15.8 | 42 | -18 | 66 |
| Cb | right | 38 | 15.7 | 21 | -69 | -33 | PoCG |  |  | *10.1* | *54* | *-15* | *57* |
| STG | right | 16 | 15.4 | 66 | -45 | 18 | BA17 | right | 34 | 15.3 | 18 | -78 | 9 |
|  |  |  |  |  |  |  | SOG | right | 17 | 13.4 | 24 | -87 | 27 |
|  |  |  |  |  |  |  | MorbG | right | 12 | 12.4 | 9 | 30 | -12 |
|  |  |  |  |  |  |  | SFG, BA6 | right | 10 | 14.5 | 18 | -15 | 72 |
|  |  |  |  |  |  |  |  |  |  |  |  |  |  |
| Abbreviations: BA, Broadman area; Cb, cerebellum; CN, caudate nucleus; IFG, inferior frontal gyrus; IPL, inferior parietal lobule; MOG, middle occipital gyrus; MorbG, medial orbital gyrus; MTG, middle temporal gyrus; Pal, pallidum; Pcl, paracentral lobule; PoCG, postcentral gyrus; PrCG, precentral gyrus; RecG, rectal gyrus; SFG, superior frontal gyrus; SMA, supplementary motor area; SmG, supramarginal gyrus; SOG, superior occipital gyrus; SPL, superior parietal lobule; STG, superior temporal gyrus; Tp, temporal pole; | | | | | | | | | | | | | |
