# Supplementary material for: Training of ultra-fast speech comprehension induces functional reorganization of the central-visual system in late-blind humans
Source: Front Hum Neurosci. 2013 Oct 23;7:701. doi: 10.3389/fnhum.2013.00701 (PMC3805979; doi:10.3389/fnhum.2013.00701)
Supplement: Supplementary file 1 — An example for forward moderately fast speech (8 syl/s). “Wegen den anstehenden wichtigen Prüfungen muss er viel lernen.” [file Presentation1.ZIP › 64044_Dietrich_Data_Sheet_6.DOCX]

| **Supplementary file 6.** Coordinates of the whole-brain analysis of all six participants arranged according to their residual vision (increasing from left to right). Hemodynamic effects of the SPM *T*-contrast “forward speech at 18 syl/s versus baseline” pre- and post-training were threshold at *p* < 0.005 at voxel level uncorrected with an extent threshold *k* = 10 voxels. If the value of the x coordinate was negative (-), the peak occurred within the left hemisphere (Coordinates describing the SPM *T*-contrast “baseline versus condition” were added as Supplementary file 7). Italicized numbers labeled sub-peaks. | | | | | | | | | | | | | | | | | | | | | | | | |
| --- | --- | --- | --- | --- | --- | --- | --- | --- | --- | --- | --- | --- | --- | --- | --- | --- | --- | --- | --- | --- | --- | --- | --- | --- |
|  | **147** | | | | **151** | | | | **150** | | | | **144** | | | | **146** | | | | **142** | | | |
| Region | T | MNI coordinate | | | T | MNI coordinate | | | T | MNI coordinate | | | T | MNI coordinate | | | T | MNI coordinate | | | T | MNI coordinate | | |
|  |  | x | y | z |  | x | y | z |  | x | y | z |  | x | y | z |  | x | y | z |  | x | y | z |
| **Pre-training** | | | | | | | | | | | | | | | | | | | | | | | | |
| ***Temporal lobe*** | | | | | | | | | | | | | | | | | | | | | | | | |
| STG | 9.5 | -60 | -21 | 6 | 14.5 | -57 | 0 | 0 | 10.5 | -63 | -9 | 6 | 15.5 | -66 | -36 | -3 | 18.1 | -60 | -15 | 3 | 17.2 | -60 | -24 | 9 |
|  | 5.5 | 63 | -24 | 0 | 16.8 | 69 | -12 | 3 | 11.3 | 66 | -24 | 9 | 15.5 | 69 | -30 | 0 | 17.6 | 60 | 0 | -6 | 16.2 | 66 | -3 | 0 |
| ITG |  |  |  |  |  |  |  |  |  |  |  |  |  |  |  |  | 3.1 | 48 | -54 | -18 |  |  |  |  |
| ***Frontal lobe*** | | | | | | | | | | | | | | | | | | | | | | | | |
| IFG |  |  |  |  |  |  |  |  | 4.8 | -39 | 12 | 27 | 5.5 | -51 | 36 | 6 |  |  |  |  | 4.0 | -45 | 12 | 27 |
|  |  |  |  |  |  |  |  |  |  |  |  |  | 4.2 | -42 | 21 | 27 |  |  |  |  |  |  |  |  |
|  |  |  |  |  |  |  |  |  | 3.2 | 33 | 18 | 27 | 8.0 | 60 | -63 | -21 | 4.2 | 45 | 6 | 27 |  |  |  |  |
|  |  |  |  |  |  |  |  |  |  |  |  |  |  |  |  |  | 4.0 | 45 | 45 | -6 |  |  |  |  |
| SMG |  |  |  |  |  |  |  |  |  |  |  |  |  |  |  |  | 3.5 | 9 | 27 | 42 |  |  |  |  |
| MFG |  |  |  |  |  |  |  |  |  |  |  |  |  |  |  |  | 3.0 | 39 | 45 | 18 |  |  |  |  |
| SMA |  |  |  |  | 3.1 | 9 | 9 | 60 |  |  |  |  |  |  |  |  |  |  |  |  |  |  |  |  |
| PrCG |  |  |  |  | 4.1 | 54 | -3 | 48 |  |  |  |  | 4.2 | -36 | -3 | 45 |  |  |  |  |  |  |  |  |
| ***Parietal lobe*** | | | | | | | | | | | | | | | | | | | | | | | | |
| PoCG |  |  |  |  |  |  |  |  | 4.0 | 45 | -39 | 60 |  |  |  |  |  |  |  |  | 3.3 | -54 | -9 | 51 |
| IPL |  |  |  |  |  |  |  |  |  |  |  |  | 3.1 | -30 | -54 | 42 | 3.6 | -54 | -33 | 51 |  |  |  |  |
| ***Others*** | | | | | | | | | | | | | | | | | | | | | | | | |
| Cb |  |  |  |  |  |  |  |  | 3.4 | -33 | -72 | -21 | 7.3 | -48 | -63 | -21 | 3.7 | -27 | -66 | -45 |  |  |  |  |
| CC |  |  |  |  |  |  |  |  |  |  |  |  |  |  |  |  | 3.4 | 3 | -27 | 30 |  |  |  |  |
|  |  |  |  |  |  |  |  |  |  |  |  |  |  |  |  |  | 2.8 | 6 | 33 | 24 |  |  |  |  |
| Ins |  |  |  |  |  |  |  |  |  |  |  |  |  |  |  |  | 3.0 | -33 | 9 | -12 |  |  |  |  |
|  |  |  |  |  |  |  |  |  |  |  |  |  |  |  |  |  | 3.5 | 36 | 27 | -3 |  |  |  |  |
| Tha |  |  |  |  |  |  |  |  | 3.0 | -18 | -24 | 0 |  |  |  |  |  |  |  |  |  |  |  |  |
| Hipp |  |  |  |  |  |  |  |  |  |  |  |  |  |  |  |  | 3.1 | -18 | -24 | -12 |  |  |  |  |
| Pal |  |  |  |  |  |  |  |  |  |  |  |  |  |  |  |  | 2.7 | -9 | 6 | -3 |  |  |  |  |
|  | | | | | | | | | | | | | | | | | | | | | | | | |
| **Post-training** | | | | | | | | | | | | | | | | | | | | | | | | |
| ***Temporal lobe*** | | | | | | | | | | | | | | | | | | | | | | | | |
| STG | 8.7 | -63 | -21 | 6 | 17.9 | -60 | -3 | 0 | 10.5 | -66 | -27 | 12 | 14.3 | -66 | -33 | -3 | 13.5 | -60 | -15 | 6 | 21.0 | -60 | -21 | 6 |
|  | 6.7 | 69 | -18 | 3 | 16.2 | 66 | -18 | 9 | 11.2 | 69 | -24 | 9 | 15.0 | 69 | -21 | -3 | 11.9 | 57 | -9 | -3 | 21.4 | 66 | -6 | 3 |
| ITG |  |  |  |  | 3.2 | -60 | -57 | -9 |  |  |  |  | 5.8 | 60 | -63 | -9 |  |  |  |  | 4.4 | 69 | -30 | -18 |
|  |  |  |  |  |  |  |  |  |  |  |  |  | *6.5* | *-63* | *-57* | *-9* |  |  |  |  |  |  |  |  |
| ***Occipital lobe*** | | | | | | | | | | | | | | | | | | | | | | | | |
| BA17/18 | 3.6 | -33 | -93 | 6 |  |  |  |  |  |  |  |  |  |  |  |  |  |  |  |  |  |  |  |  |
|  | 5.0 | 27 | -96 | 12 | 3.7 | 18 | -93 | 3 | 3.0 | 3 | -84 | 3 |  |  |  |  |  |  |  |  |  |  |  |  |
| SOG /  MOG | 4.1 | -24 | -96 | 18 | 3.3 | -15 | -84 | 18 |  |  |  |  |  |  |  |  |  |  |  |  |  |  |  |  |
|  | 4.6 | 42 | -87 | 0 |  |  |  |  |  |  |  |  |  |  |  |  |  |  |  |  |  |  |  |  |
| FG | 4.7 | -39 | -69 | -15 |  |  |  |  |  |  |  |  |  |  |  |  |  |  |  |  |  |  |  |  |
|  | 3.3 | 39 | -54 | -12 |  |  |  |  |  |  |  |  |  |  |  |  |  |  |  |  |  |  |  |  |
| ***Frontal lobe*** | | | | | | | | | | | | | | | | | | | | | | | | |
| IFG | 2.9 | -51 | 18 | 0 | 4.3 | -60 | 21 | 21 | 6.8 | -39 | 12 | 27 | *6.4* | *-51* | *33* | *9* | *6.8* | *-66* | *-27* | *-18* | *13.2* | *-48* | *12* | *27* |
|  |  |  |  |  |  |  |  |  |  |  |  |  | 4.5 | 48 | 33 | 0 |  |  |  |  | 5.0 | 57 | 27 | 3 |
| MFG |  |  |  |  | 3.5 | 39 | 9 | 54 |  |  |  |  | 3.9 | 48 | -3 | 54 |  |  |  |  | 3.2 | -33 | 42 | 15 |
| OrbG |  |  |  |  |  |  |  |  | 3.5 | 15 | 45 | -15 | 3.4 | 24 | 48 | -18 |  |  |  |  |  |  |  |  |
| SMG |  |  |  |  |  |  |  |  | 3.2 | -6 | 33 | 48 |  |  |  |  |  |  |  |  | 4.6 | -9 | 36 | 60 |
| SMA |  |  |  |  |  |  |  |  | 4.0 | -3 | -3 | 66 | 3.9 | -3 | 0 | 60 |  |  |  |  | 7.2 | -6 | 3 | 63 |
| PrCG |  |  |  |  |  |  |  |  | 3.4 | -6 | 57 | -9 | 4.5 | -36 | 3 | 48 |  |  |  |  |  |  |  |  |
|  |  |  |  |  |  |  |  |  | 3.0 | -42 | -9 | 39 | 4.2 | -21 | -9 | 78 |  |  |  |  |  |  |  |  |
|  |  |  |  |  |  |  |  |  |  |  |  |  | 2.8 | -39 | -21 | 60 |  |  |  |  |  |  |  |  |
|  |  |  |  |  |  |  |  |  |  |  |  |  | 3.6 | 30 | -21 | 63 |  |  |  |  |  |  |  |  |
| ***Parietal lobe*** | | | | | | | | | | | | | | | | | | | | | | | | |
| PoCG | 3.2 | 39 | -45 | 63 |  |  |  |  | 3.8 | 57 | -3 | 21 |  |  |  |  |  |  |  |  |  |  |  |  |
| PcL |  |  |  |  |  |  |  |  |  |  |  |  |  |  |  |  |  |  |  |  | 4.6 | -9 | -30 | 78 |
| Prc |  |  |  |  |  |  |  |  |  |  |  |  |  |  |  |  |  |  |  |  | 4.6 | -6 | -72 | 39 |
| SPL | 4.0 | 33 | -72 | 48 |  |  |  |  |  |  |  |  |  |  |  |  |  |  |  |  | 7.4 | -33 | -48 | 69 |
|  |  |  |  |  |  |  |  |  |  |  |  |  |  |  |  |  |  |  |  |  | 3.3 | -33 | -63 | 48 |
| IPL |  |  |  |  |  |  |  |  |  |  |  |  |  |  |  |  | 3.0 | -48 | -54 | 48 | 7.2 | -63 | -27 | 39 |
|  |  |  |  |  | 3.8 | 36 | -51 | 45 |  |  |  |  |  |  |  |  | 2.9 | 57 | -48 | 39 |  |  |  |  |
| AG / IPL |  |  |  |  |  |  |  |  |  |  |  |  |  |  |  |  | 3.7 | 42 | -60 | 51 |  |  |  |  |
| ***Other*** | | | | | | | | | | | | | | | | | | | | | | | | |
| Cb | *3.7* | *-27* | *-51* | *-18* | 4.1 | -18 | -90 | -24 |  |  |  |  | 5.8 | -48 | -63 | -21 | 3.1 | -6 | -87 | -21 | 5.3 | -12 | -78 | -39 |
|  |  |  |  |  | 3.1 | -24 | -60 | -18 | 3.7 | 24 | -63 | -48 | 3.7 | 30 | -63 | -51 | 3.0 | -36 | -84 | -30 | 7.7 | 15 | -78 | -42 |
|  |  |  |  |  |  |  |  |  |  |  |  |  |  |  |  |  |  |  |  |  | 4.5 | 30 | -63 | -51 |
|  |  |  |  |  |  |  |  |  |  |  |  |  |  |  |  |  |  |  |  |  | 4.2 | 33 | -66 | -24 |
|  |  |  |  |  |  |  |  |  |  |  |  |  |  |  |  |  |  |  |  |  | 4.3 | 42 | -51 | -30 |
| CC |  |  |  |  | 3.2 | 9 | 27 | 27 | 3.7 | 6 | 18 | 45 |  |  |  |  |  |  |  |  |  |  |  |  |
| CN |  |  |  |  | 3.0 | 12 | 12 | 12 |  |  |  |  |  |  |  |  |  |  |  |  |  |  |  |  |
| Tha |  |  |  |  | 2.9 | 12 | -12 | 9 |  |  |  |  |  |  |  |  |  |  |  |  | 5.9 | -6 | -15 | 9 |
|  |  |  |  |  |  |  |  |  |  |  |  |  |  |  |  |  |  |  |  |  |  |  |  |  |
| Abbreviations: AG, angular gyrus; BA, Brodman area; Cb, cerebellum; CC, cingulate cortex; CN, caudate nucleus; FG, fusiform gyrus; Hipp, hippocampus; IFG, inferior frontal gyrus; Ins, insula; IPL, inferior parietal lobe; ITG, inferior temporal gyrus; MFG, medial frontal gyrus; OrbG, orbital gyrus; Pal, pallidum; PcL, paracentral lobe; PoCG, postcentralgyrus; PrCG, precentralgyrus; Prc, precuneus; SMA, supplementary motor area; SMG, superior medial gyrus; MOG, middle occipital gyrus; SOG, superior occipital gyrus; SPL, superior parietal lobe; STG, superior temporal gyrus; Tha, thalamus. | | | | | | | | | | | | | | | | | | | | | | | | |
