# Supplementary material for: Training of ultra-fast speech comprehension induces functional reorganization of the central-visual system in late-blind humans
Source: Front Hum Neurosci. 2013 Oct 23;7:701. doi: 10.3389/fnhum.2013.00701 (PMC3805979; doi:10.3389/fnhum.2013.00701)
Supplement: Supplementary file 1 — An example for forward moderately fast speech (8 syl/s). “Wegen den anstehenden wichtigen Prüfungen muss er viel lernen.” [file Presentation1.ZIP › 64044_Dietrich_Data_Sheet_7.DOCX]

| **Supplementary file 7.** Coordinates of the whole-brain analysis of all six participants arranged according to their residual vision (increasing from left to right). Hemodynamic effects of the SPM *T*-contrast “baseline versus forward speech at 18 syl/s” pre- and post-training were threshold at *p* < 0.005 at voxel level uncorrected with an extent threshold *k* = 10 voxels. If the value of the x coordinate was negative (-), the peak occurred within the left hemisphere. Italicized numbers labeled sub-peaks. | | | | | | | | | | | | | | | | | | | | | | | | | | | | |
| --- | --- | --- | --- | --- | --- | --- | --- | --- | --- | --- | --- | --- | --- | --- | --- | --- | --- | --- | --- | --- | --- | --- | --- | --- | --- | --- | --- | --- |
|  | **147** | | | | **151** | | | | **150** | | | | **144** | | | | | | | **146** | | | | | **142** | | | |
| Region | T | MNI coordinate | | | T | MNI coordinate | | | T | MNI coordinate | | | T | | MNI coordinate | | | | | T | | MNI coordinate | | | T | MNI coordinate | | |
|  |  | x | y | z |  | x | y | z |  | x | y | z |  | | x | | y | z | |  | | x | y | z |  | x | y | z |
| **Pre-training** | | | | | | | | | | | | | | | | | | | | | | | | | | | | |
| ***Occipital lobe*** | | | | | | | | | | | | | | | | | | | | | | | | | | | | |
| Prc | 3.0 | -31 | -81 | 45 |  |  |  |  |  |  |  |  | 3.4 | | -12 | | -48 | 63 | | 3.18 | | -9 | -63 | 60 | 3.4 | 3 | -54 | 57 |
| CalcG |  |  |  |  | 2.7 | -15 | -51 | 12 |  |  |  |  |  | |  | |  |  | |  | |  |  |  | 3.5 | 27 | -54 | 9 |
| BA17, 18 |  |  |  |  |  |  |  |  |  |  |  |  | 3.0 | | -9 | | -60 | 0 | | *3.2* | | *-15* | *-51* | *0* | 4.4 | -15 | -93 | 24 |
|  |  |  |  |  |  |  |  |  |  |  |  |  |  | |  | |  |  | |  | |  |  |  | 2.9 | -21 | -69 | -12 |
|  |  |  |  |  |  |  |  |  |  |  |  |  |  | |  | |  |  | |  | |  |  |  | 3.0 | 12 | -87 | -9 |
| MOG |  |  |  |  |  |  |  |  |  |  |  |  | 3.7 | | -36 | | -84 | 21 | |  | |  |  |  |  |  |  |  |
| IOG |  |  |  |  |  |  |  |  |  |  |  |  |  | |  | |  |  | |  | |  |  |  | 4.0 | -48 | -78 | -3 |
| FG |  |  |  |  |  |  |  |  |  |  |  |  | 3.8 | | 27 | | -39 | -18 | |  | |  |  |  | 3.1 | 30 | -54 | -15 |
|  |  |  |  |  |  |  |  |  |  |  |  |  | 2.8 | | -24 | | -81 | -18 | |  | |  |  |  | 3.4 | -27 | -48 | -15 |
| ***Temporal lobe*** | | | | | | | | | | | | | | | | | | | | | | | | | | | | |
| ITG |  |  |  |  | 3.4 | -51 | -51 | -9 |  |  |  |  |  | |  | |  |  | |  | |  |  |  | 3.6 | -54 | -54 | -9 |
| Tp |  |  |  |  | 3.9 | -51 | 18 | -18 |  |  |  |  |  | |  | |  |  | |  | |  |  |  |  |  |  |  |
| MTG/AG |  |  |  |  | 4.1 | -33 | -66 | 42 | 3.4 | 48 | -69 | 21 | 5.2 | | 48 | | -72 | 30 | |  | |  |  |  |  |  |  |  |
| ***Parietal lobe*** | | | | | | | | | | | | | | | | | | | | | | | | | | | | |
| SmG |  |  |  |  | 3.8 | -60 | -21 | 39 |  |  |  |  | 3.6 | | 57 | | -33 | 42 | |  | |  |  |  |  |  |  |  |
| IPL |  |  |  |  | 3.6 | -39 | -48 | 48 |  |  |  |  | 2.9 | | -51 | | -36 | 36 | |  | |  |  |  | *3.7* | *-54* | *-39* | *36* |
| SPL |  |  |  |  | 3.2 | 33 | -72 | 51 |  |  |  |  |  | |  | |  |  | |  | |  |  |  |  |  |  |  |
| PoCG |  |  |  |  |  |  |  |  |  |  |  |  | 4.2 | | 21 | | -33 | 78 | |  | |  |  |  | 4.2 | -57 | -18 | 33 |
|  |  |  |  |  |  |  |  |  |  |  |  |  |  | |  | |  |  | |  | |  |  |  | 3.4 | -30 | -36 | 60 |
|  |  |  |  |  |  |  |  |  |  |  |  |  |  | |  | |  |  | |  | |  |  |  | 4.6 | 69 | -12 | 27 |
| ***Frontal lobe*** | | | | | | | | | | | | | | | | | | | | | | | | | | | | |
| IFG |  |  |  |  | 3.0 | -39 | 24 | 15 |  |  |  |  |  | |  | |  |  | |  | |  |  |  | *3.4* | *-48* | *24* | *21* |
|  |  |  |  |  |  |  |  |  |  |  |  |  |  | |  | |  |  | |  | |  |  |  | 4.7 | -60 | 9 | 9 |
|  |  |  |  |  |  |  |  |  |  |  |  |  |  | |  | |  |  | |  | |  |  |  | 4.0 | 54 | 15 | 0 |
| MFG |  |  |  |  | 3.4 | -30 | 27 | 45 |  |  |  |  | 3.8 | | -30 | | 39 | 39 | |  | |  |  |  | 4.2 | -42 | 27 | 39 |
|  |  |  |  |  | 2.9 | -36 | 60 | 3 |  |  |  |  | 5.8 | | 24 | | 24 | 39 | |  | |  |  |  | 3.8 | 33 | 30 | 33 |
|  |  |  |  |  | 2.8 | -39 | 45 | 15 |  |  |  |  |  | |  | |  |  | |  | |  |  |  | 3.0 | 27 | 54 | 24 |
| SFG |  |  |  |  |  |  |  |  |  |  |  |  | 3.1 | | 24 | | 9 | 69 | |  | |  |  |  | *3.2* | *15* | *15* | *51* |
| PrCG |  |  |  |  | 4.3 | -51 | 3 | 39 |  |  |  |  |  | |  | |  |  | |  | |  |  |  | 3.1 | -33 | -6 | 63 |
|  |  |  |  |  |  |  |  |  |  |  |  |  |  | |  | |  |  | |  | |  |  |  | 3.2 | 57 | 3 | 42 |
| SMA |  |  |  |  |  |  |  |  |  |  |  |  | 3.4 | | -3 | | 18 | 72 | |  | |  |  |  | 4.0 | -12 | 9 | 66 |
|  |  |  |  |  |  |  |  |  |  |  |  |  |  | |  | |  |  | |  | |  |  |  | 3.6 | 12 | 0 | 69 |
| SorG |  |  |  |  |  |  |  |  |  |  |  |  | 4.0 | | 27 | | 36 | -12 | |  | |  |  |  |  |  |  |  |
| ***Other*** | | | | | | | | | | | | | | | | | | | | | | | | | | | | |
| Cb |  |  |  |  |  |  |  |  |  |  |  |  | 3.5 | | -9 | | -51 | -51 | |  | |  |  |  | *3.0* | *-36* | *-48* | *-27* |
|  |  |  |  |  |  |  |  |  |  |  |  |  |  | |  | |  |  | |  | |  |  |  | 3.1 | -21 | -57 | -57 |
|  |  |  |  |  |  |  |  |  |  |  |  |  |  | |  | |  |  | |  | |  |  |  | 3.1 | 30 | -45 | 66 |
| CC |  |  |  |  | 3.4 | -6 | -33 | 39 |  |  |  |  |  | |  | |  |  | |  | |  |  |  | 3.6 | -6 | -12 | 42 |
| CC |  |  |  |  | 3.0 | 15 | 36 | 15 |  |  |  |  | 3.5 | | 9 | | 36 | 15 | |  | |  |  |  | 4.1 | -3 | 9 | 30 |
| Ins |  |  |  |  |  |  |  |  |  |  |  |  |  | |  | |  |  | |  | |  |  |  | 3.0 | -39 | -3 | 9 |
|  | | | | | | | | | | | | | | | | | | | | | | | | | | | | |
| **Post-training** | | | | | | | | | | | | | | | | | | | | | | | | | | | | |
| ***Temporal lobe*** | | | | | | | | | | | | | | | | | | | | | | | | | | | | |
| MTG |  |  |  |  |  |  |  |  |  |  |  |  | 4.0 | -60 | | -3 | | | -24 | | 3.7 | -51 | -66 | 15 | 5.0 | 66 | -15 | -15 |
|  |  |  |  |  |  |  |  |  |  |  |  |  |  |  | |  | | |  | | 3.8 | 54 | -57 | 9 |  |  |  |  |
| ITG |  |  |  |  |  |  |  |  |  |  |  |  | 4.5 | -57 | | -21 | | | -24 | |  |  |  |  | 4.7 | -63 | -21 | -24 |
|  |  |  |  |  |  |  |  |  |  |  |  |  | 3.5 | 60 | | -24 | | | -27 | |  |  |  |  | 3.8 | 54 | -30 | -18 |
|  |  |  |  |  |  |  |  |  |  |  |  |  | 3.4 | -36 | | 9 | | | -39 | |  |  |  |  |  |  |  |  |
| Tp |  |  |  |  |  |  |  |  |  |  |  |  | 4.1 | 45 | | 18 | | | -27 | |  |  |  |  |  |  |  |  |
| ***Occipital lobe*** | | | | | | | | | | | | | | | | | | | | | | | | | | | | |
| BA17/18 |  |  |  |  |  |  |  |  |  |  |  |  | 3.1 | -15 | | -87 | | | -15 | | 3.1 | 24 | -54 | -9 | 3.4 | 12 | -72 | -6 |
|  |  |  |  |  |  |  |  |  |  |  |  |  |  |  | |  | | |  | | 3.1 | -12 | -81 | 0 |  |  |  |  |
|  |  |  |  |  |  |  |  |  |  |  |  |  | 3.5 | 9 | | -90 | | | 30 | | 3.8 | -21 | -48 | -9 |  |  |  |  |
|  |  |  |  |  |  |  |  |  |  |  |  |  | 3.1 | -6 | | -102 | | | 12 | | 3.2 | 9 | -87 | 18 |  |  |  |  |
|  |  |  |  |  |  |  |  |  |  |  |  |  |  |  | |  | | |  | | 3.2 | 21 | -87 | -6 |  |  |  |  |
| IOG |  |  |  |  |  |  |  |  |  |  |  |  | 2.9 | -54 | | -72 | | | -6 | |  |  |  |  |  |  |  |  |
| MOG | 3.3 | -36 | -87 | 18 |  |  |  |  |  |  |  |  |  |  | |  | | |  | |  |  |  |  |  |  |  |  |
| SOG |  |  |  |  |  |  |  |  |  |  |  |  |  |  | |  | | |  | | 3.6 | -12 | -84 | 42 |  |  |  |  |
| Prc |  |  |  |  |  |  |  |  | 3.1 | -6 | -72 | 51 |  |  | |  | | |  | | 4.2 | -6 | -60 | 63 |  |  |  |  |
| FG | 3.8 | -42 | -54 | -21 |  |  |  |  |  |  |  |  | 3.7 | -27 | | -45 | | | -9 | |  |  |  |  |  |  |  |  |
|  |  |  |  |  |  |  |  |  |  |  |  |  | 2.9 | 27 | | -45 | | | -15 | |  |  |  |  |  |  |  |  |
| ***Frontal lobe*** | | | | | | | | | | | | | | | | | | | | | | | | | | | | |
| IFG |  |  |  |  |  |  |  |  |  |  |  |  | 6.3 | -48 | | 39 | | | -6 | |  |  |  |  | 4.8 | -45 | 45 | 15 |
|  |  |  |  |  |  |  |  |  |  |  |  |  | 4.4 | -27 | | 18 | | | -24 | |  |  |  |  |  |  |  |  |
| SFG |  |  |  |  |  |  |  |  |  |  |  |  | 3.9 | -21 | | 18 | | | 66 | |  |  |  |  | 6.7 | 24 | 21 | 57 |
|  |  |  |  |  |  |  |  |  |  |  |  |  | 3.0 | -12 | | 27 | | | 54 | |  |  |  |  |  |  |  |  |
| MFG |  |  |  |  |  |  |  |  | 3.2 | 39 | 57 | 24 | 4.8 | -27 | | 48 | | | 33 | | 3.3 | -21 | 6 | 57 | 7.6 | -39 | 27 | 48 |
|  |  |  |  |  |  |  |  |  |  |  |  |  | 4.4 | 27 | | 57 | | | 24 | |  |  |  |  |  |  |  |  |
|  |  |  |  |  |  |  |  |  |  |  |  |  | 4.9 | 45 | | 45 | | | 9 | |  |  |  |  |  |  |  |  |
|  |  |  |  |  |  |  |  |  |  |  |  |  | 3.7 | 33 | | 24 | | | 42 | |  |  |  |  |  |  |  |  |
|  |  |  |  |  |  |  |  |  |  |  |  |  | 4.1 | -36 | | 18 | | | 42 | |  |  |  |  |  |  |  |  |
|  |  |  |  |  |  |  |  |  |  |  |  |  | 3.6 | -39 | | 39 | | | 27 | |  |  |  |  |  |  |  |  |
| SMG |  |  |  |  |  |  |  |  |  |  |  |  |  |  | |  | | |  | |  |  |  |  | 4.2 | 6 | 63 | 0 |
| SorG |  |  |  |  |  |  |  |  |  |  |  |  | 3.6 | -12 | | 60 | | | -6 | |  |  |  |  |  |  |  |  |
| MorG |  |  |  |  |  |  |  |  |  |  |  |  | 4.1 | 42 | | 30 | | | -15 | |  |  |  |  |  |  |  |  |
| PrCG |  |  |  |  |  |  |  |  |  |  |  |  | 3.6 | 57 | | 0 | | | 42 | | 3.1 | 39 | -15 | 42 | 3.8 | 24 | -21 | 78 |
| ***Parietal lobe*** | | | | | | | | | | | | | | | | | | | | | | | | | | | | |
| AG |  |  |  |  |  |  |  |  |  |  |  |  |  |  | |  | | |  | |  |  |  |  | 8.1 | 36 | -75 | 42 |
| SmG |  |  |  |  |  |  |  |  |  |  |  |  | 6.8 | 63 | | -27 | | | 33 | |  |  |  |  | 6.0 | -66 | -27 | 27 |
| PoCG |  |  |  |  |  |  |  |  |  |  |  |  |  |  | |  | | |  | | 3.3 | -24 | -42 | 57 | 3.8 | 57 | -9 | 24 |
|  |  |  |  |  |  |  |  |  |  |  |  |  |  |  | |  | | |  | |  |  |  |  | 3.4 | -30 | -33 | 63 |
|  |  |  |  |  |  |  |  |  |  |  |  |  |  |  | |  | | |  | |  |  |  |  | 3.3 | -57 | -18 | 42 |
| IPL | 3.4 | -30 | -75 | 51 |  |  |  |  |  |  |  |  | 6.6 | -60 | | -42 | | | 39 | |  |  |  |  |  |  |  |  |
|  |  |  |  |  |  |  |  |  |  |  |  |  |  |  | |  | | |  | |  |  |  |  |  |  |  |  |
| ***Other*** | | | | | | | | | | | | | | | | | | | | | | | | | | | | |
| CC |  |  |  |  |  |  |  |  |  |  |  |  |  |  | |  | | |  | |  |  |  |  | 4.1 | 6 | -27 | 48 |
| Cb | 4.4 | 45 | -78 | -24 |  |  |  |  |  |  |  |  |  |  | |  | | |  | |  |  |  |  | 5.0 | -48 | -45 | -39 |
|  | 4.0 | 36 | -42 | -39 |  |  |  |  |  |  |  |  |  |  | |  | | |  | |  |  |  |  | 3.7 | 36 | -78 | -33 |
|  | 3.4 | -27 | -90 | -24 |  |  |  |  |  |  |  |  |  |  | |  | | |  | |  |  |  |  |  |  |  |  |
|  | 4.2 | -45 | -75 | -39 |  |  |  |  |  |  |  |  |  |  | |  | | |  | |  |  |  |  |  |  |  |  |
| CN |  |  |  |  |  |  |  |  | 2.6 | 6 | 15 | -6 |  |  | |  | | |  | |  |  |  |  |  |  |  |  |
| Put |  |  |  |  |  |  |  |  |  |  |  |  | 5.3 | 24 | | 12 | | | -6 | |  |  |  |  |  |  |  |  |
|  |  |  |  |  |  |  |  |  |  |  |  |  | *3.4* | *-27* | | *3* | | | *-3* | |  |  |  |  |  |  |  |  |
| Tha |  |  |  |  |  |  |  |  |  |  |  |  | 3.6 | 9 | | -12 | | | 9 | |  |  |  |  |  |  |  |  |
|  |  |  |  |  |  |  |  |  |  |  |  |  |  |  | |  | | |  | |  |  |  |  |  |  |  |  |
| Abbreviations: AG, angular gyrus; BA, Brodman area; CalcG, calcarine gyrus; Cb, cerebellum; CC, cingulate cortex; CN, caudate nucleus; FG, fusiform gyrus; IFG, inferior frontal gyrus; Ins, insula; IOG, inferior occipital gyrus; IPL, inferior parietal lobe; ITG, inferior temporal gyrus; MFG, medial frontal gyrus; MOG, middle occipital gyrus; MorG, middle orbital gyrus; PoCG, postcentralgyrus; PrCG, precentralgyrus; Prc, precuneus; Put, putamen; SFG, superior frontal gyrus; SorG, superior orbital gyrus; SMA, supplementary motor area; SMG, superior medial gyrus; SmG, supramarginal gyrus; SOG, superior occipital gyrus; SPL, superior parietal lobe; Tha, thalamus; Tp, temporal pole. | | | | | | | | | | | | | | | | | | | | | | | | | | | | |
