# Supplementary material for: Training of ultra-fast speech comprehension induces functional reorganization of the central-visual system in late-blind humans
Source: Front Hum Neurosci. 2013 Oct 23;7:701. doi: 10.3389/fnhum.2013.00701 (PMC3805979; doi:10.3389/fnhum.2013.00701)
Supplement: Supplementary file 1 — An example for forward moderately fast speech (8 syl/s). “Wegen den anstehenden wichtigen Prüfungen muss er viel lernen.” [file Presentation1.ZIP › 64044_Dietrich_Data_Sheet_8.DOCX]

| **Supplementary file 8.** Coordinates of the whole-brain analysis of all six participants arranged according to their residual vision (increasing from left to right). Hemodynamic effects of the SPM *T*-contrast “post- versus pre-training during forward speech at 18 syl/s” were threshold at *p* < 0.005 at voxel level uncorrected with contiguous voxels *k* = 10. If the value of the x coordinate was negative (-), the peak occurred within the left hemisphere. Italicized numbers labeled sub-peaks. | | | | | | | | | | | | | | | | | | | | | | | | |
| --- | --- | --- | --- | --- | --- | --- | --- | --- | --- | --- | --- | --- | --- | --- | --- | --- | --- | --- | --- | --- | --- | --- | --- | --- |
|  | **147** | | | | **151** | | | | **150** | | | | **144** | | | | **146** | | | | **142** | | | |
| Region | T | MNI coordinate | | | T | MNI coordinate | | | T | MNI coordinate | | | T | MNI coordinate | | | T | MNI coordinate | | | T | MNI coordinate | | |
|  |  | x | y | z |  | x | y | z |  | x | y | z |  | x | y | z |  | x | y | z |  | x | y | z |
|  | | | | | | | | | | | | | | | | | | | | | | | | |
| **Post- minus pre-training (forward ultra-fast speech)** | | | | | | | | | | | | | | | | | | | | | | | | |
| ***Temporal lobe*** | | | | | | | | | | | | | | | | | | | | | | | | |
| MTG | 4.4 | -69 | -33 | 9 |  |  |  |  |  |  |  |  | 3.8 | -51 | -30 | -9 |  |  |  |  | 4.3 | -60 | -54 | 0 |
|  |  |  |  |  |  |  |  |  |  |  |  |  | 4.5 | -60 | -9 | -6 |  |  |  |  | 4.1 | 48 | -21 | -9 |
|  |  |  |  |  |  |  |  |  |  |  |  |  |  |  |  |  |  |  |  |  | 3.3 | 63 | -45 | 6 |
|  |  |  |  |  |  |  |  |  |  |  |  |  |  |  |  |  |  |  |  |  | 4.0 | -51 | -21 | -9 |
| STG | *4.0* | *-57* | *-45* | *12* | *3.7* | *-63* | *0* | *-3* |  |  |  |  | *3.5* | *-57* | *3* | *-9* |  |  |  |  |  |  |  |  |
|  | *4.6* | *72* | *-21* | *3* |  |  |  |  |  |  |  |  |  |  |  |  |  |  |  |  |  |  |  |  |
| ITG | *3.7* | *-48* | *-27* | *-24* |  |  |  |  |  |  |  |  |  |  |  |  |  |  |  |  |  |  |  |  |
|  | 4.7 | 54 | -30 | -21 |  |  |  |  |  |  |  |  |  |  |  |  |  |  |  |  |  |  |  |  |
|  | 3.1 | -60 | -48 | -18 |  |  |  |  |  |  |  |  |  |  |  |  |  |  |  |  |  |  |  |  |
| Tp | *2.9* | *-54* | *9* | *-15* | 5.6 | -57 | 9 | -9 |  |  |  |  | 3.6 | -54 | 12 | -6 |  |  |  |  |  |  |  |  |
|  | 3.5 | 57 | 15 | -21 |  |  |  |  |  |  |  |  |  |  |  |  |  |  |  |  |  |  |  |  |
| ***Occipital lobe*** | | | | | | | | | | | | | | | | | | | | | | | | |
| FG | 3.6 | -36 | -66 | -12 |  |  |  |  |  |  |  |  |  |  |  |  |  |  |  |  |  |  |  |  |
|  | 3.1 | -24 | -51 | -15 |  |  |  |  |  |  |  |  |  |  |  |  |  |  |  |  |  |  |  |  |
| BA18 | 3.1 | 18 | -93 | 15 |  |  |  |  |  |  |  |  |  |  |  |  |  |  |  |  |  |  |  |  |
|  | 3.2 | -15 | -96 | 9 |  |  |  |  |  |  |  |  |  |  |  |  |  |  |  |  |  |  |  |  |
| BA17 | *3.0* | *18* | *-81* | *12* |  |  |  |  |  |  |  |  |  |  |  |  |  |  |  |  |  |  |  |  |
| MOG | 3.0 | -27 | -72 | 33 |  |  |  |  |  |  |  |  |  |  |  |  |  |  |  |  |  |  |  |  |
|  | 3.0 | -45 | -72 | 9 |  |  |  |  |  |  |  |  |  |  |  |  |  |  |  |  |  |  |  |  |
| SOG | *2.9* | *-18* | *-84* | *24* |  |  |  |  |  |  |  |  |  |  |  |  |  |  |  |  |  |  |  |  |
|  | 3.3 | 33 | -69 | 42 |  |  |  |  |  |  |  |  |  |  |  |  |  |  |  |  |  |  |  |  |
| IOG | 3.5 | -33 | -81 | -3 |  |  |  |  |  |  |  |  |  |  |  |  |  |  |  |  |  |  |  |  |
| ***Frontal lobe*** | | | | | | | | | | | | | | | | | | | | | | | | |
| RecG | 4.6 | -12 | 27 | -12 | *3.3* | *3* | *33* | *-15* |  |  |  |  |  |  |  |  |  |  |  |  |  |  |  |  |
| PrCG | *3.6* | *-39* | *-18* | *57* | 4.0 | -54 | 0 | 45 |  |  |  |  | 2.8 | 30 | -18 | 63 |  |  |  |  | *4.4* | *-51* | *9* | *30* |
|  | 3.0 | 45 | -15 | 60 |  |  |  |  |  |  |  |  |  |  |  |  |  |  |  |  |  |  |  |  |
|  | 3.0 | -36 | 0 | 36 |  |  |  |  |  |  |  |  |  |  |  |  |  |  |  |  |  |  |  |  |
| IFG | 3.2 | -48 | 18 | 0 | 3.1 | -42 | 30 | 9 |  |  |  |  | 2.9 | 51 | 24 | 3 |  |  |  |  | 6.7 | -54 | 21 | -3 |
|  |  |  |  |  |  |  |  |  |  |  |  |  | *2.9* | *-45* | *18* | *-3* |  |  |  |  | *4.8* | *57* | *27* | *3* |
| MFG |  |  |  |  | *2.9* | *-18* | *30* | *36* |  |  |  |  | 4.0 | -39 | 54 | 21 |  |  |  |  |  |  |  |  |
|  |  |  |  |  | 3.0 | 45 | 48 | 0 |  |  |  |  | 3.3 | 27 | 39 | 33 |  |  |  |  |  |  |  |  |
|  |  |  |  |  |  |  |  |  |  |  |  |  | 3.1 | 36 | 54 | 18 |  |  |  |  |  |  |  |  |
| SMA |  |  |  |  |  |  |  |  |  |  |  |  | 3.2 | -12 | 0 | 63 |  |  |  |  | *4.4* | *-3* | *9* | *54* |
|  |  |  |  |  |  |  |  |  |  |  |  |  | *2.8* | *12* | *-12* | *75* |  |  |  |  |  |  |  |  |
| SFG |  |  |  |  |  |  |  |  |  |  |  |  | 2.8 | 15 | -15 | 72 |  |  |  |  |  |  |  |  |
| ***Parietal lobe*** | | | | | | | | | | | | | | | | | | | | | | | | |
| PoCG | 3.6 | -39 | -33 | 51 | *3.6* | *-57* | *-9* | *48* |  |  |  |  |  |  |  |  |  |  |  |  |  |  |  |  |
| SmG | 3.5 | -45 | -42 | 27 |  |  |  |  |  |  |  |  |  |  |  |  |  |  |  |  | 5.9 | -57 | -21 | 21 |
| AG |  |  |  |  | 3.7 | -42 | -66 | 36 |  |  |  |  |  |  |  |  |  |  |  |  |  |  |  |  |
|  |  |  |  |  | *3.0* | *54* | *-60* | *51* |  |  |  |  |  |  |  |  |  |  |  |  |  |  |  |  |
| IPL |  |  |  |  | *3.3* | *-42* | *-51* | *51* |  |  |  |  |  |  |  |  |  |  |  |  | 4.1 | -57 | -51 | 39 |
|  |  |  |  |  | 3.0 | 57 | -48 | 51 |  |  |  |  |  |  |  |  |  |  |  |  |  |  |  |  |
| Prc |  |  |  |  | 3.2 | 3 | -75 | 48 |  |  |  |  |  |  |  |  |  |  |  |  |  |  |  |  |
| ***Other*** | | | | | | | | | | | | | | | | | | | | | | | | |
| Cb | *3.5* | *-21* | *-66* | *-21* |  |  |  |  |  |  |  |  |  |  |  |  |  |  |  |  |  |  |  |  |
|  | 3.3 | 24 | -69 | -21 |  |  |  |  |  |  |  |  |  |  |  |  |  |  |  |  |  |  |  |  |
|  | 3.0 | 9 | -84 | -39 |  |  |  |  |  |  |  |  |  |  |  |  |  |  |  |  |  |  |  |  |
| PhippG | 3.2 | 30 | 0 | -30 |  |  |  |  |  |  |  |  |  |  |  |  |  |  |  |  |  |  |  |  |
| Amg | 3.0 | -18 | 0 | -21 |  |  |  |  |  |  |  |  |  |  |  |  |  |  |  |  |  |  |  |  |
| CC |  |  |  |  | 3.8 | 9 | 27 | 30 |  |  |  |  | 3.5 | 6 | 18 | 39 |  |  |  |  | 3.2 | 6 | -15 | 30 |
|  |  |  |  |  | 3.1 | -9 | 27 | 27 |  |  |  |  |  |  |  |  |  |  |  |  | 4.7 | -3 | 30 | 24 |
| Bstem |  |  |  |  |  |  |  |  |  |  |  |  |  |  |  |  | 3.3 | 6 | -42 | -54 |  |  |  |  |
| Ins |  |  |  |  |  |  |  |  |  |  |  |  |  |  |  |  |  |  |  |  | 4.8 | 39 | 21 | -6 |
| Pal |  |  |  |  |  |  |  |  |  |  |  |  |  |  |  |  |  |  |  |  | 3.7 | -21 | 0 | -3 |
| Hipp |  |  |  |  | 3.5 | -27 | -33 | -3 |  |  |  |  |  |  |  |  |  |  |  |  |  |  |  |  |
|  | | | | | | | | | | | | | | | | | | | | | | | | |
| **Pre- minus post-training (forward ultra-fast speech)** | | | | | | | | | | | | | | | | | | | | | | | | |
| ***Temporal lobe*** | | | | | | | | | | | | | | | | | | | | | | | | |
| MTG |  |  |  |  | 4.0 | 54 | -60 | 9 |  |  |  |  | 4.2 | -60 | 0 | -24 | 2.9 | -51 | -69 | 6 | 3.5 | -63 | -9 | -6 |
|  |  |  |  |  |  |  |  |  |  |  |  |  | 4.6 | -66 | -42 | -6 | 2.8 | 45 | -60 | 9 |  |  |  |  |
|  |  |  |  |  |  |  |  |  |  |  |  |  | 3.1 | -60 | -18 | -18 |  |  |  |  |  |  |  |  |
| STG |  |  |  |  | 8.9 | 69 | -12 | 3 |  |  |  |  |  |  |  |  | 6.3 | 48 | -33 | 12 | 5.3 | 69 | -21 | 12 |
|  |  |  |  |  | 3.5 | 69 | -30 | 18 |  |  |  |  |  |  |  |  | 6.9 | -54 | -21 | 3 | 3.8 | -48 | -30 | 9 |
|  |  |  |  |  | 3.2 | -60 | -12 | 3 |  |  |  |  |  |  |  |  |  |  |  |  |  |  |  |  |
|  |  |  |  |  | 3.3 | -54 | -30 | 6 |  |  |  |  |  |  |  |  |  |  |  |  |  |  |  |  |
|  |  |  |  |  | 3.6 | -51 | 0 | -3 |  |  |  |  |  |  |  |  |  |  |  |  |  |  |  |  |
| HG |  |  |  |  |  |  |  |  | 4.0 | 42 | -21 | 6 |  |  |  |  |  |  |  |  |  |  |  |  |
| ***Occipital lobe*** | | | | | | | | | | | | | | | | | | | | | | | | |
| FG |  |  |  |  | 3.2 | -39 | -51 | -12 |  |  |  |  |  |  |  |  |  |  |  |  |  |  |  |  |
| SOG |  |  |  |  |  |  |  |  |  |  |  |  | 3.0 | 21 | -99 | 21 |  |  |  |  |  |  |  |  |
| ***Frontal lobe*** | | | | | | | | | | | | | | | | | | | | | | | | |
| PrCG |  |  |  |  | 3.3 | 54 | -6 | 51 |  |  |  |  |  |  |  |  |  |  |  |  |  |  |  |  |
| SMA |  |  |  |  | 3.0 | 9 | 9 | 60 |  |  |  |  |  |  |  |  |  |  |  |  |  |  |  |  |
| IFG |  |  |  |  |  |  |  |  |  |  |  |  | 4.6 | -51 | 39 | -6 |  |  |  |  |  |  |  |  |
| MFG |  |  |  |  |  |  |  |  |  |  |  |  | 3.8 | 39 | 36 | 42 |  |  |  |  | 3.9 | -33 | 21 | 51 |
|  |  |  |  |  |  |  |  |  |  |  |  |  |  |  |  |  |  |  |  |  | 4.0 | 24 | 33 | 42 |
|  |  |  |  |  |  |  |  |  |  |  |  |  |  |  |  |  |  |  |  |  | 3.0 | 30 | 54 | 6 |
| SFG |  |  |  |  |  |  |  |  |  |  |  |  | 3.2 | -12 | 54 | 30 |  |  |  |  | 3.4 | 33 | 0 | 66 |
|  |  |  |  |  |  |  |  |  |  |  |  |  |  |  |  |  |  |  |  |  | 3.5 | -15 | 45 | 39 |
| ***Parietal lobe*** | | | | | | | | | | | | | | | | | | | | | | | | |
| AG |  |  |  |  |  |  |  |  |  |  |  |  | 4.8 | -51 | -69 | 24 |  |  |  |  | 4.0 | 36 | -75 | 42 |
|  |  |  |  |  |  |  |  |  |  |  |  |  | 3.8 | 42 | -66 | 54 |  |  |  |  |  |  |  |  |
| SmG |  |  |  |  |  |  |  |  |  |  |  |  | 3.7 | 63 | -24 | 33 |  |  |  |  | 6.3 | -66 | -27 | 27 |
|  |  |  |  |  |  |  |  |  |  |  |  |  | 3.1 | 63 | -36 | 45 |  |  |  |  |  |  |  |  |
| SPL |  |  |  |  |  |  |  |  |  |  |  |  |  |  |  |  | 3.0 | 24 | -66 | 54 |  |  |  |  |
| Prc |  |  |  |  |  |  |  |  |  |  |  |  |  |  |  |  |  |  |  |  | 4.4 | -9 | -51 | 42 |
| ***Other*** | | | | | | | | | | | | | | | | | | | | | | | | |
| Cb |  |  |  |  | 3.5 | -30 | -60 | -48 |  |  |  |  |  |  |  |  | 3.2 | 33 | -69 | -48 |  |  |  |  |
| Hipp |  |  |  |  |  |  |  |  |  |  |  |  |  |  |  |  | 3.5 | -21 | -27 | -12 |  |  |  |  |
|  |  |  |  |  |  |  |  |  |  |  |  |  |  |  |  |  |  |  |  |  |  |  |  |  |
| Abbreviations: AG, angular gyrus; Amg, amygdala; BA, Brodman area; Bstem, brain-stem; Cb, cerebellum; CC, cingulate cortex; FG, fusiform gyrus; HG, heschl’s gyrus; Hipp, hippocampus; IFG, inferior frontal gyrus; Ins, insula; IOG, inferior occipital gyrus; IPL, inferior parietal lobe; ITG, inferior temporal gyrus; MFG, medial frontal gyrus; MOG, middle occipital gyrus; MTG, middle temporal gyrus; Pal, pallidum; PhippG, parahippocampal gyrus; PoCG, postcentral gyrus; PrCG, precentral gyrus; Prc, precuneus; RecG, rectal gyrus; SFG, superior frontal gyrus; SMA, supplementary motor area; SmG, supramarginal gyrus; SOG, superior occipital gyrus; SPL, superior parietal lobe; STG, superior temporal gyrus; Tp, temporal pole. | | | | | | | | | | | | | | | | | | | | | | | | |
